# Current Nutritional Approaches to Maintain and Improve Broiler Meat Quality: Part-2 Functional Feed Additives

**DOI:** 10.3390/ani16203165

**Published:** 2026-10-09

**Authors:** Tarek A. Ebeid, Emad F. Mousa, Abdullah N. Alkhalaf

**Affiliations:** 1 Department of Animal and Poultry Production, College of Agriculture and Food, Qassim University, P.O. Box 6622, Buraidah 51452, Saudi Arabia; 2 Department of Pathology & Laboratory Diagnosis, College of Veterinary Medicine, Qassim University, Buraidah 51452, Saudi Arabia

**Keywords:** chickens, meat, probiotics, prebiotics, synbiotics, organic acids, phytobiotics, trace minerals, vitamins, natural antioxidants

## Abstract

The worldwide consumption of poultry meat is expected to increase due to its high protein content, low fat concentration, and ease of digestion. Numerous functional feed additives, including probiotics, prebiotics, synbiotics, organic acids, phytobiotics, natural antioxidants, vitamins, and trace minerals, have gained great attention due to their impact on performance, gut health, immunity, and meat quality. Therefore, the objective of the present review is to critically evaluate the role of functional feed additives in improving meat physicochemical quality, nutritional value, and oxidative stability in broilers. Probiotics and prebiotics consistently improved water-holding capacity and reduced cooking loss and shear force, whereas their effects on pH and color were more variable across studies, and they enhanced the meat’s nutritional profile by increasing polyunsaturated fatty acids and lowering saturated fatty acids. Although the effects of synbiotics and organic acids on physical traits can be inconsistent, they effectively modified the fatty acid profile toward essential healthy fats and improved the oxidative stability of meat. Phytobiotics and natural antioxidants (such as polyphenols and flavonoids) improved water-holding capacity and tenderness as well as suppressed meat lipid oxidation through the activation of antioxidative enzymes and the nuclear factor erythroid 2-related factor 2 (*Nrf2*) signaling pathway. Furthermore, the incorporation of trace elements like Se, Zn, and Mn in the form of organic minerals or nanoparticles improved physical meat characteristics, nutritional value, oxidative stability, and shelf life of meat. Notably, combined or matrix-based strategies (such as synbiotic formulations, organic acid–phytogenic blends, and vitamin–mineral matrices) are increasingly used in commercial poultry nutrition and, in several of the studies reviewed, produced favorable effects on meat quality; however, with the exception of studies employing a dose-titrated or factorial design, the cited literature generally did not include a direct single-component comparator arm, so whether these combinations act synergistically rather than additively remains to be formally established. It can be concluded that functional feed additives, whether applied singly or in strategic combination, provided several benefits for the poultry industry by enhancing physicochemical properties of meat and reducing biochemical markers of oxidative deterioration, outcomes consistent with, though not a direct substitute for, extended shelf life of meat.

## Simple Summary

Poultry meat is widely consumed because it is a nutritious, affordable, and easily digestible source of protein. Improving its quality and nutritional value is therefore important for consumers and the poultry industry. This review summarizes how functional feed additives, including probiotics, prebiotics, synbiotics, organic acids, plant-based additives, natural antioxidants, vitamins, and trace minerals, can improve poultry meat physicochemical quality, nutritional value, and oxidative stability. These additives can consistently enhance meat tenderness and its ability to retain water, whereas their effects on meat acidity (pH) and color are more variable and depend on the additive type, dose, and chemical form. They may also improve the nutritional value of meat by increasing beneficial fatty acids and reducing less desirable saturated fats. Importantly, several of these additives—particularly plant-based additives, natural antioxidants, and trace minerals—protect meat from chemical deterioration caused by oxidation, a key determinant of post-slaughter meat quality retention and a major challenge in commercial poultry production, as reflected by reduced biochemical markers of lipid and protein oxidation. Although some additives, particularly synbiotics and organic acids, show variable effects on certain meat characteristics, their overall potential for improving meat quality, nutritional value, and oxidative stability is promising. The use of functional feed additives may therefore provide practical benefits for poultry producers by improving meat physicochemical quality, nutritional composition, and oxidative stability under the conditions examined in the reviewed studies. Claims regarding economic return, extended shelf life in the strict sense, or human health outcomes were beyond the scope of the primary studies reviewed and are not asserted here.

## 1. Introduction

Across nations and religions, poultry meat, especially that from chickens, is highly regarded for its flavor, high protein level, low fat concentration, and ease of digestion, leading to an increase in its consumption compared with red meat [1]. Nutritionally, poultry meat is considered nutritious and healthy food because it offers high-quality protein comprising all essential amino acids, vitamins (niacin, vitamin B6, and vitamin B12), and minerals (phosphorus, selenium, and zinc) [2,3]. However, the sustained genetic selection for rapid growth rate and high breast muscle yield that has underpinned the commercial success of the broiler industry has concurrently outpaced the muscle’s physiological capacity for adequate vascularization, oxidative metabolism, and structural maturation, creating a set of quality challenges that now confront the industry at a market-relevant scale [4]. Foremost among these are the emerging breast muscle myopathies (including wooden breast, white striping, and spaghetti meat), which are increasingly documented in fast-growing broiler lines and are associated with localized muscle hypoxia, an inflammatory and oxidative microenvironment, degeneration of muscle fibers, and fibrotic/lipidotic replacement of contractile tissue [5]. These myopathies not only compromise the visual and textural acceptability of fresh cuts but also downgrade the economic value of affected carcasses, as processors increasingly reject or discount myopathy-affected breast filets destined for premium retail and food-service channels. Moreover, poultry muscle is prone to oxidative damage due to its high polyunsaturated fatty acids (PUFAs) content, active myoglobin/heme-iron turnover, and fast metabolism from rapid growth. After slaughter, with antioxidant regeneration stopped, reactive oxygen species (ROS) degrade membrane lipids and proteins [6]. This causes discoloration, rancidity, reduced water-holding capacity (WHC), and shorter shelf life, leading to problems that hurt distribution, increase waste, and erode consumer trust, especially as cold-chain retail and export reliance grows [7].

The physicochemical properties of meat are key factors of its quality, palatability, and consumer acceptance. These properties include pH, WHC, color [including lightness (L*), redness (a*), and yellowness (b*) values], tenderness, drip loss, cooking loss, shear force, and chemical composition such as moisture, crude protein (CP), fat (ether extract, EE), and ash content. These properties influence meat storage stability, processing efficiency, and consumer decisions regarding meat quality and meat products [6]. Sensory traits (cohesiveness, hardness, juiciness, chewiness, fibrosity, and flavor) are important for consumers’ preferences [8]. Meat chemical composition consists of water, protein, fat, minerals, and vitamins. The approximate composition of broiler chicken meat varies according to meat cut, tissue type, and other factors, but poultry meat is generally characterized by a high moisture and protein content and relatively low-fat content in lean cuts [9]. Chicken meat quality characteristics are affected by genetic and non-genetic factors, including breed, sex, age, nutrition, housing, management, environmental temperature, stress, processing, and storage [6].

**Figure 1:**
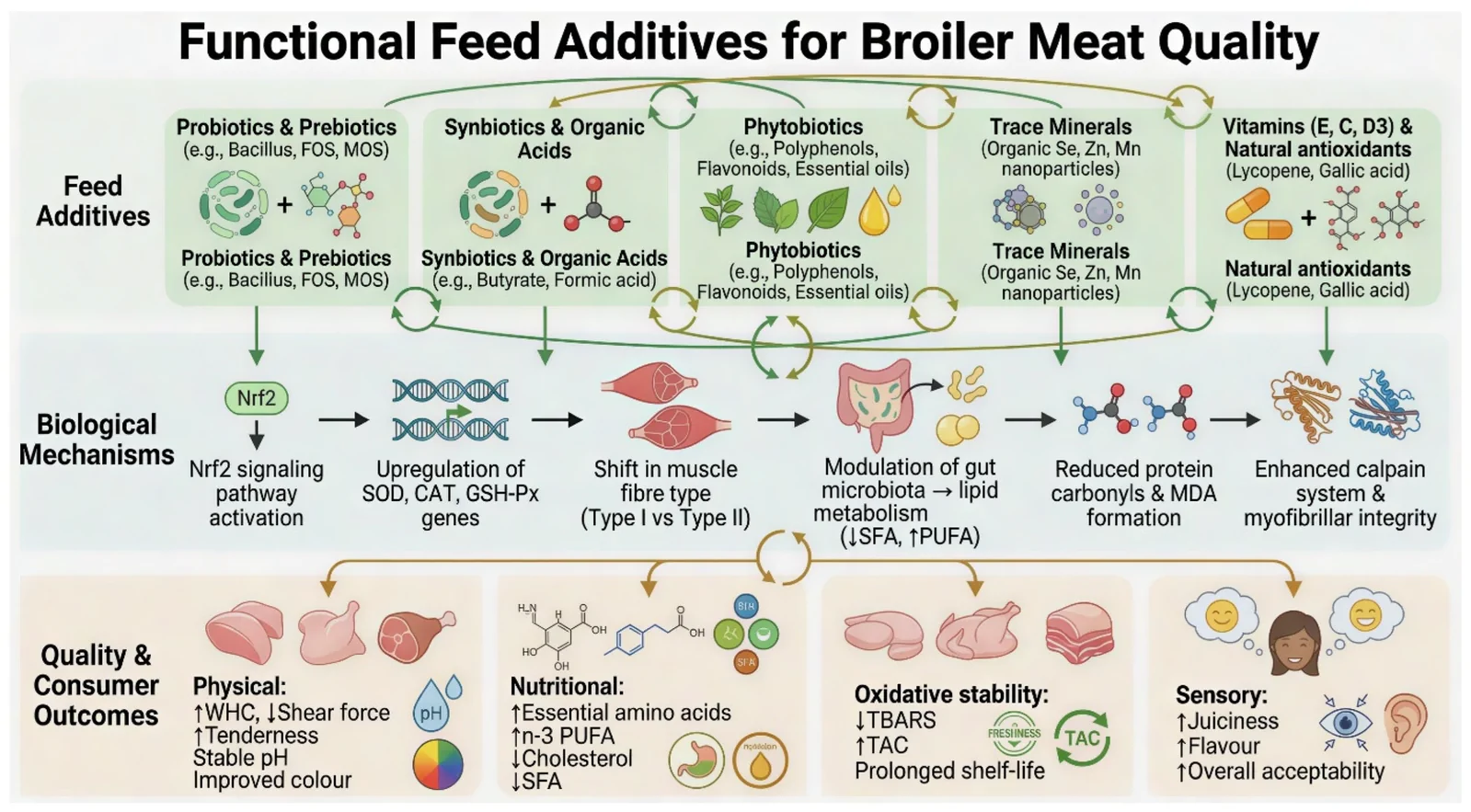
Summary of the impacts of functional feed additives on meat quality traits in broilers (This figure was created by the authors using Microsoft PowerPoint and BioRender.com).

In recent years, numerous functional feed additives, including probiotics, prebiotics, synbiotics, organic acids, phytobiotics, natural antioxidants, vitamins, and trace minerals, have gained great attention due to their impact on performance, gut microbiota modulation, immunity, inflammatory signaling, muscle protein turnover, lipid metabolism, and meat quality (Figure 1). These functional feed additives offer a mechanistically grounded route to mitigating oxidative muscle damage, supporting muscle structural integrity, and extending postmortem shelf life, while simultaneously improving the nutritional value of the resulting meat. Several recent reviews have addressed related aspects of poultry nutrition and meat quality, but each differs from the present work in scope or emphasis. Choi et al. [10] reviewed nutritional strategies for improving broiler meat quality and composition specifically under challenging production conditions (e.g., heat stress, high stocking density, and fast growth-related myopathies), rather than evaluating functional feed additives as a standalone nutritional category under standard rearing conditions. Samad et al. [11] examined functional feed additives primarily through the lens of antibiotic reduction, addressing meat quality alongside food safety, antimicrobial resistance, economic feasibility, and consumer perception, but without the additive-by-additive, dose-referenced synthesis of physicochemical, nutritional, and oxidative stability outcomes provided here. Niu et al. [12] offered a broader mechanistic synthesis spanning both dietary macronutrient manipulation (protein, amino acids, fats) and functional additives within a single narrative organized around the gut–muscle axis and muscle fiber conversion. Also, Sampath and Kim [13] reviewed functional feed additives in the context of sustainable broiler production, with an emphasis on production sustainability and performance outcomes, rather than providing the additive-by-additive, dose-referenced synthesis of physicochemical, nutritional, and oxidative stability outcomes in meat that is the focus of the present review. In contrast to these works, the present review (i) is restricted to broilers, (ii) integrates eight functional feed additive categories (probiotics, prebiotics, synbiotics, organic acids, phytobiotics, trace minerals, vitamins, and natural antioxidants) within a single, physicochemically and nutritionally organized framework, and (iii) explicitly separates functional feed additive strategies (Part 2) from diet-composition and feeding-management strategies (Part 1, companion review), a scope broader than any single one of the works cited above. Therefore, the objective of the present review (Part 2) is to emphasize the recent results linked to the role of functional feed additives (namely probiotics, prebiotics, synbiotics, organic acids, phytobiotics, trace minerals, vitamins, and natural antioxidants) in improving or maintaining meat quality characteristics and its nutritional value (Figure 1). This review builds on the companion Part 1, which addressed diet manipulation and feeding-management strategies (crude protein and amino acid balancing, dietary lipid and omega-3 sources, exogenous enzyme supplementation, feed restriction, and pre-slaughter fasting), reflecting a deliberate division between diet/feeding-regime-based strategies (Part 1) and additive-based strategies (Part 2) in modulating broiler meat quality.

## 2. Materials and Methods

A structured literature search was conducted to identify relevant scientific publications addressing functional feed additives affecting broiler meat quality. The literature search was performed using PubMed, Scopus, Web of Science, and Google Scholar. The search focused primarily on recent peer-reviewed studies published during the last five years, while selected earlier publications were retained when they provided important foundational information or established concepts relevant to the topic. Combinations of keywords and Boolean operators were used, including broiler chickens, poultry meat quality, functional feed additives, probiotics, prebiotics, synbiotics, organic acids, phytobiotics, phytogenic feed additives, essential oils, trace minerals, nanoparticles, vitamins, natural antioxidants, polyphenols, flavonoids, physicochemical properties, meat composition, fatty acid profile, and oxidative stability. Retrieved publications were initially screened based on their titles and abstracts, followed by full-text assessment when considered relevant to the scope of the review. Priority was given to original peer-reviewed studies that directly investigated the effects of dietary probiotics, prebiotics, synbiotics, organic acids, phytobiotics, trace minerals, vitamins, or natural antioxidants on physicochemical characteristics, chemical composition, fatty acid profile, sensory attributes, or oxidative stability of broiler meat. Studies that were not directly related to broiler meat quality or did not provide relevant information on functional feed additives were excluded. Particular emphasis was placed on recent studies to reflect current developments in poultry nutrition, whereas older studies were included when required to provide fundamental or supporting information. A total of 92 scientific journal articles were included in the present review. In addition, two authoritative or complementary sources—a book chapter and an OECD/FAO report—were consulted to provide supporting nutritional and background information, resulting in a total of 94 references cited in the manuscript. The selected literature was critically evaluated and synthesized according to the major topics addressed in this review, including probiotics, prebiotics, synbiotics, organic acids, phytobiotics or phytogenic feed additives, trace minerals, vitamins, and natural antioxidants.

## 3. Probiotics

Probiotics are live micro-organisms used as an alternative to antibiotics and play a vital role in gut development, nutrient utilization, gut microbiota, antioxidative status, immune responsiveness, and productive performance in poultry [14]. Recent reports have documented that dietary probiotics are a safe and promising strategy to improve the quality of poultry meat [15,16,17]. Dietary probiotic supplementation affected meat physical characteristics [15,16,18] and chemical composition [18,19,20]. Mohammed et al. [15] elucidated that the addition of *Bacillus subtilis* PB6 supplementation (2 × 10^7^ CFU/g) increased L*, a*, and b* values at 30 min and 5 h post-mortem, improved WHC, enhanced sensory measurements (flavor, texture, preference, and general aspect) at 5 h after slaughter, reduced cooking loss, and minimized pH at 5 h post-mortem in broiler leg muscle. Wang et al. [16] observed that the addition of *Bacillus subtilis* (3.2 × 10^9^ CFU/g) enhanced muscular pH, color, WHC, and shear force. These outcomes are mechanistically interconnected rather than independent traits. A lower ultimate pH reflects more rapid or extensive postmortem glycolysis and lactate accumulation, which in turn accelerates myofibrillar protein denaturation, shrinks the space between thick and thin filaments, and reduces the number of polar groups available to bind water. Furthermore, one of the most important mechanistic explanations for these improvements is the probiotic-induced shift in muscle fiber composition and metabolic activity. Wang et al. [16] demonstrated that dietary *Bacillus subtilis* increased the proportion of oxidative (type I) muscle fibers and reduced the proportion of glycolytic (type II) muscle fibers in thigh muscle through activation of AMP-activated protein kinase (AMPK), silent information regulator 1 (SIRT1), and peroxisome proliferator-activated receptor gamma coactivator 1 alpha (PGC-1α). Whether this AMPK/SIRT1/PGC-1α-mediated fiber-type shift represents a conserved mode of action across other probiotic strains and genera remains to be determined, and future studies employing muscle fiber typing and gene expression analyses across a broader range of probiotic products are needed to establish the generalizability of this mechanism. Al-Abdullatif et al. [17] reported that the addition of a probiotic mixture containing *Bacillus subtilis* (1 × 10^9^ CFU/mL), *Lactobacillus parabunchneri* (1 × 10^9^ CFU/mL), *Saccharomyces cerevisiae* yeast (1 × 10^5^ CFU/mL), *Lactobacillus harbinensis* (1 × 10^9^ CFU/mL), *Rhodopseudomonas palustris* (1 × 10^7^ CFU/mL), *Rhodopseudomonas sphaeroides* (1 × 10^7^ CFU/mL), and *Candida ethanolica* (1 × 10^5^ CFU/mL) had a positive effect on ultimate pH (pH_24h_), cooking loss, shear force, texture profile (hardness, springiness, cohesiveness, and chewiness), and sensory properties (juiciness and acceptability) in the breast muscle of broilers. It should be noted that this study did not include single-strain treatment arms, so the relative contribution of individual components, and whether the observed effects reflect a synergistic interaction among the seven microbial components rather than the action of one or a few dominant strains, cannot be determined from this experimental design. Liu et al. [19] noted that dietary inclusion of *Brevibacillus laterosporus* S62-9 (1 × 10^9^ CFU/g) reduced pH, L* value, pressing loss, cooking loss, and shear force, whilst a* value and sensory traits (aroma, tenderness, and springiness) were increased in the breast meat. Similarly, probiotics including *Bacillus subtilis* (2 × 10^9^ spores/g, [18]) and *Bacillus pumilus* SA388 (0.8 mg/kg, [20]), as an antibiotic alternative, positively influence juiciness, tenderness, aroma, taste, color, drip loss, and cooking loss in the breast muscle of broilers. The improvements in WHC and texture profile might be linked with muscle protein functionality, particularly the ability of myofibrillar proteins to retain water as well as protect cells from oxidative damage [21]. Several studies pointed out that dietary probiotics enhanced sensory attributes, including aroma, flavor, preference, acceptability, and general appearance [15,17,19]. Taken together, it might be indicated that the addition of probiotics had a beneficial effect on meat quality characteristics, including color, WHC, cooking loss, shear force, texture profile parameters, and sensory characteristics in the breast and thigh muscles of broilers (Table 1). It is worth noting that the direction of the pH response specifically diverged between studies. Wang et al. [16] reported that dietary *Bacillus subtilis* (3.2 × 10^9^ CFU/g) elevated muscular pH, whereas Liu et al. [19] reported that *Brevibacillus laterosporus* S62-9 (1 × 10^9^ CFU/g) reduced pH in the breast meat. This divergence may plausibly be related to differences in the bacterial genus and its associated postmortem metabolic activity (*Bacillus* versus *Brevibacillus*), rather than representing a generalized or strain-independent probiotic effect on postmortem glycolysis.

Probiotics may also positively affect poultry meat chemical composition (moisture, CP, EE, and crude ash), enhancing the concentration of total PUFAs and decreasing the content of total saturated fatty acids (SFAs) in meat. Beyari et al. [20] showed that supplementing broiler diets with *Bacillus pumilus* SA388 (0.8 mg/kg) increased breast meat contents of moisture and protein, but reduced fat and ash contents compared to the control. Liu et al. [19] noted that the dietary addition of *Brevibacillus laterosporus* S62-9 (1 × 10^9^ CFU/g) increased protein content in the breast meat and fat content in thigh meat of broilers. Also, breast meat amino acid composition (serine, threonine, alanine, methionine, phenylalanine, arginine, and total essential amino acids) and volatile compound profile (about a 20-fold rise in the amounts of hexanal and 1-octen-3-ol) increased. Similarly, Tang et al. [18] observed that dietary supplementation of *Bacillus subtilis* (2 × 10^9^ spores/g) had no negative effect on meat chemical composition (moisture, CP, EE, and crude ash), while breast muscle concentration of lysine, methionine, glutamic acid, total essential amino acids, and glutamic acid in thigh muscle was higher than in the control group. Also, dietary supplementation of *Bacillus subtilis* increased breast muscle content of palmitoleic acid (C16:1n-7), oleic acid (C18:1), Cis-8,11,14-eicosatrienoate, total monounsaturated fatty acids (MUFAs), eicosadienoic acid (20:2n-6), total unsaturated fatty acids (UFAs), and total PUFAs, but reduced its content of palmitic acid (C16:0), arachidic acid (C20:0), and total SFAs in *Pectoralis* muscle. Similarly, Eglite et al. [22] explained that *Lactobacillus farciminis* and *Lactobacillus rhamnosus* (4 g/10 kg of feed) elevated the concentrations of n-3 PUFAs and n-6 PUFAs in the breast meat and decreased concentrations of SFAs in the breast meat of broilers. These improvements in fatty acid profile might be due to the fact that probiotics can modulate key genes involved in lipid metabolism, including acetyl-CoA carboxylase alpha (ACCα), sterol regulatory element-binding protein-1c (SREBP-1c), fatty acid synthase, and stearoyl-CoA desaturase 1 mRNA [23]. It should be noted that direct molecular evidence for this specific mechanism in broiler skeletal muscle is currently limited; the cited gene-expression pathway was characterized in Sunit sheep, in which probiotic supplementation increased intramuscular fat and modified fatty acid composition via the AMPK signaling pathway [23]. While the involvement of these lipogenic regulatory genes in mediating fatty acid profile changes is biologically plausible and consistent with conserved lipid metabolism pathways across livestock species, this mechanism should be regarded as a hypothesis extrapolated from ruminant evidence rather than a confirmed pathway in poultry, and broiler-specific transcriptomic or gene-expression studies are needed to validate its applicability to chicken skeletal muscle. It might indicate that incorporating probiotics into broilers’ diets significantly enhanced the nutritional profile and chemical composition of breast and thigh meat.

Meat oxidative stability is the resistance of meat to oxidation that deteriorates its quality by altering color, flavor, freshness, and nutritional value. One major factor contributing to the deterioration of food quality is lipid oxidation, which produces unpleasant flavors and odors [15,16]. The thiobarbituric acid reactive substances (TBARS) assessment is the most popular test for determining malondialdehyde (MDA) concentrations in tissues and assessing the result of reduced antioxidant defense against free radicals in meat [16,20]. Using TBARS as a measure of lipid oxidation, several published studies found that dietary probiotics reduced meat lipid oxidation [16,20,24]. Mohammed et al. [15] elucidated that the addition of *Bacillus subtilis* PB6 supplementation (2 × 10^7^ CFU/g) enhanced meat oxidative stability by enhancing total antioxidant capacity (TAC) and reducing protein oxidation (protein carbonyls) in the breast meat. Beyari et al. [20] showed that supplementing broiler diets with *Bacillus pumilus* SA388 (0.8 mg/kg) reduced breast meat lipid peroxidation (2-thiobarbituric acid) in comparison with the control. Wang et al. [16] elucidated that the addition of *Bacillus subtilis* (3.2 × 10^9^ CFUs/g) enhanced the activities of CAT, SOD, glutathione peroxidase (GSH-Px), and TAC, while lessening the MDA level in thigh muscle. They also noted that dietary *Bacillus subtilis* supplementation elevated the gene and protein expression of nuclear factor erythroid 2-related factor 2 (*Nrf2*) and activated the Nrf2 signaling pathway, leading to improved oxidative stability of meat. Additional support for probiotic-mediated Nrf2 pathway activation as a general antioxidative mechanism comes from Lu et al. [25], who reported that *Bacillus coagulans* HYI alleviated lipopolysaccharide-induced oxidative stress via the Nrf2/HO-1 signaling pathway in laying hens; although this study assessed systemic oxidative stress and gut microbiota modulation rather than meat quality outcomes, and was conducted in a different poultry production category (laying hens vs. broilers), it lends cross-context physiological support to the notion that Nrf2 pathway activation is a conserved antioxidative response to probiotic supplementation across chicken production types. Collectively, it is noteworthy to indicate that dietary probiotic supplementation might minimize oxidative damage in breast and thigh meat and maximize antioxidative properties via the Nrf2 pathway, leading to greater oxidative stability of these muscles, a biochemical outcome consistent with, though distinct from, directly measured shelf-life endpoints (Table 2). It is worth noting that several of these probiotic strains simultaneously increased meat PUFA content (see fatty acid discussion above), which would theoretically be expected to raise susceptibility to lipid peroxidation; however, the parallel enhancement of antioxidant enzyme activity and TBARS/MDA reduction reported in these studies indicates that probiotics act synergistically on both traits rather than improving nutritional quality at the expense of oxidative stability. Similar coupling between increased PUFA content and enhanced antioxidant defense is also apparent for prebiotics, organic acids, and trace minerals discussed in Section 4, Section 6, and Section 8, respectively.

**Table 1:** Effects of functional feed additives on physical meat quality traits in broilers.

Nutritional Strategy	Representative Commercial/Chemical Examples and Inclusion Levels	Traits with Consistent Directional Effect	Variable or Inconsistent Effects	Proposed Physiological Basis	References
**Probiotics**	*Bacillus subtilis*, 2 × 10^9^ spores/g diet; *Bacillus subtilis*, 3.2 × 10^9^ CFU/g diet	Improved WHC. Reduced cooking loss and shear force.	Effects on pH and color were inconsistent	Improved muscle fiber characteristics, antioxidant status, and postmortem metabolism	[16,18]
**Prebiotics**	Fructo-oligosaccharides, 0.5% of diet; mannan-oligosaccharides, 0.2% of diet; gum Arabic, 0.12–0.75% of diet	Reduced shear force and cooking loss.	Effects on pH and color were inconsistent	Enhanced gut health and antioxidative protection	[26,27,28]
**Synbiotics**	*Lactobacillus acidophilus* + mannan-oligosaccharides (1 × 10^6^ CFU/g + 0.2%); *L. casei* + *L. acidophilus* + *Bifidobacterium animalis* + inulin/chicory fiber, 1 g/kg diet	Improved WHC. Reduced drip loss	Variable effects on color and pH	Synergistic modulation of gut microbiota and nutrient utilization	[29,30,31]
**Organic acids**	Sodium butyrate, 1 g/kg diet; coated sodium butyrate, 250–1000 mg/kg; mixed organic acid blend (formic, acetic, propionic, lactic, sorbic, citric acids), 3000–6000 mg/kg	Reduced drip and cooking losses in several studies	Some studies reported negligible effects	Improved intestinal health and altered lipid metabolism	[8,32]
**Phytobiotics**	Essential oil blend (oregano, cinnamon, orange peel extracts), 250 mg/L drinking water; phytogenic additive (turmeric, red pepper, white mustard, sweet flag, soapwort), 100 ppm	Improved WHC, tenderness, and color stability.	Responses depended on plant source and dosage	Bioactive phytochemicals and antioxidant effects	[33,34]
**Organic minerals and nanoparticles**	Biogenic SeNPs, 5 mg/kg; Zn amino acid complex, 50 mg/kg; amino acid-chelated Fe/Cu/Zn/Mn premix, 300–500 mg/kg	Improved pH stability. Reduced drip loss.	Variable effects on color and tenderness	Enhanced mineral bioavailability and antioxidative defense	[35,36]
**Vitamins**	Vitamin E, 150 mg/kg diet; vitamin A, 5000–46,000 IU/kg diet; vitamin D3, 3600–5000 IU/kg diet	Vitamin E consistently improved WHC and reduced drip loss	Effects of vitamins A and D3 on color were directionally inconsistent across studies	Protection of cellular and sarcolemmal membranes from oxidative damage	[37,38,39]
**Natural antioxidants**	Lycopene, 200 mg/kg diet; epigallocatechin gallate, 750 mg/kg diet; gallic acid, 450 mg/kg diet	Improved a* value Reduced cooking loss and drip loss	Effects on pH and L* value varied among compounds	Polyphenol-mediated antioxidant activity and modulation of tenderness-related gene expression	[40,41,42]

**Table 2:** Effects of functional feed additives on meat nutritional value and oxidative stability in broilers.

Nutritional Strategy	Representative Commercial/Chemical Examples and Inclusion Levels	Main Impacts on Meat Composition and Fatty Acids	Main Impacts on Oxidative Stability	Main Molecular/Physiological Mechanisms	References
**Probiotics**	*Bacillus subtilis*, 2 × 10^9^ spores/g; *B. subtilis* KG109, 1.8 × 10^9^ CFU/kg	Increased CP, amino acids, PUFAs. Reduced SFAs.	Reduced TBARS/MDA. Enhanced antioxidant enzymes.	Activation of AMPK and Nrf2 pathways	[16,18]
**Prebiotics**	Polysaccharides of *Morinda officinalis*, 500–1000 mg/kg; fructo-oligosaccharides, 0.5% of diet	Reduced fat and cholesterol. Increased PUFAs and amino acids	Enhanced TAC. Reduced lipid oxidation.	Upregulation of antioxidant-related genes	[27,28]
**Synbiotics**	*Lactobacillus acidophilus* + mannan-oligosaccharides, 1 × 10^6^ CFU/g + 0.2%; *Bifidobacterium bifidum* + mannan-oligosaccharides, 1 × 10^6^–10^7^ CFU/g + 0.2%	Improved fatty acid profile and protein content	Reduced lipid peroxidation. Enhanced antioxidant capacity.	Synergistic modulation of redox status and lipid metabolism	[29,30]
**Organic acids**	Sodium butyrate, 1 g/kg diet; microencapsulated organic acid–bioactive compound blend (calcium formate, citric/fumaric acid, orange essential oil, thymol, carvacrol), 150–300 g/t	Improved PUFA/SFA ratio. Reduced free fatty acids.	Enhanced antioxidant activity and shelf life.	Regulation of lipid metabolism and oxidative balance	[7,32]
**Phytobiotics**	Essential oil blend (oregano, cinnamon, orange peel), 250 mg/L drinking water; epigallocatechin gallate, 750 mg/kg diet	Improved fatty acid composition and amino acid profile.	Increased SOD, CAT, and GSH-Px activities. Reduced MDA.	Polyphenol-mediated antioxidant defense and gene regulation	[43]
**Organic minerals and nanoparticles**	SeNPs, 0.4 mg/kg diet; Mn amino acid complex, 40–160 mg/kg diet	Increased protein and n-3 PUFA contents. Reduced fat content.	Enhanced antioxidant enzyme activity. Reduced TBARS.	Improved mineral bioavailability and antioxidant gene expression	[44,45]
**Vitamins**	Vitamin E, 200 IU/kg + vitamin C, 250 mg/kg + organic Se, 0.2 mg/kg (combined); vitamin D3, 5000 IU/kg diet	Minor effects on proximate composition and fatty acid profile	Reduced MDA Elevated tissue α-tocopherol and Se concentrations	Membrane protection and free radical scavenging	[32,46]
**Natural antioxidants**	Olive oil byproduct extract rich in hydroxytyrosol, 22.5 mg/kg + vitamin E, 10 mg/kg; epigallocatechin gallate, 750 mg/kg diet	Improved essential and non-essential amino acid profile	Reduced lipid peroxidation Enhanced SOD and CAT activities	Polyphenol-mediated antioxidant defense and gene regulation (SOD mRNA upregulation)	[41,47]

## 4. Prebiotics

Prebiotics can be defined as non-digestible substances that accelerate the growth of beneficial microbiota in the gut and improve its health. Several studies concluded that dietary prebiotics had a positive effect on meat quality characteristics [26,27,48]. Yan et al. [28] observed that dietary supplementation of 0.5% fructo-oligosaccharides increased pH and b* value and decreased shear force of broilers’ breast meat and increased hardness, fracturability, gumminess, and chewiness of cooked thigh meat. Also, Wu et al. [27] reported that the addition of polysaccharides of *Morinda officinalis* (1000 mg/kg) enhanced the a* value of *Pectoralis* muscle, while reducing shear force and muscle fibers’ cross-sectional area and diameter in the breast and thigh meat of Wenchang chickens. In addition, Wenchang chicken is a Chinese indigenous, slow-growing dual-purpose breed with inherently smaller muscle fiber diameter, lower growth rate, and higher intramuscular fat content relative to fast-growing commercial broiler hybrids. So, these breed-specific traits may partly account for the observed changes in muscle fiber architecture and should be considered when extrapolating the polysaccharide’s efficacy to genetically distinct, fast-growing broiler strains. These results suggest that prebiotic supplementation may influence muscle fiber development and architecture in the form of reducing muscle fiber size, leading to improved meat texture and tenderness. The beneficial effects of prebiotics on meat quality might be attributed to improving intestinal barrier integrity. Fermentation of fructo-oligosaccharides, mannan-oligosaccharides, and plant polysaccharides by beneficial gut microbiota generates short-chain fatty acids (SCFAs, principally butyrate, propionate, and acetate), which serve as the primary energy substrate for colonocytes/enterocytes, upregulate tight-junction protein expression (occludin, claudins, zonula occludens-1), and increase villus height-to-crypt depth ratio. This tightened paracellular barrier limits luminal antigen and endotoxin translocation, reducing the low-grade gut-derived inflammatory and oxidative burden that would otherwise divert metabolic resources away from muscle accretion. The resulting improvement in nutrient and amino acid absorptive capacity, rather than gut health as a generic concept, provides the direct physiological link between prebiotic fermentation, gut barrier function, and the improved protein deposition and muscle fiber architecture reported by Wu et al. [27] and Al-Baadani et al. [26]. Namted et al. [49] elucidated that administration of fructo-oligosaccharides (0.25% and 0.50%) in drinking water decreased cooking loss in the breast meat of broiler chickens. However, fructo-oligosaccharides had no negative effect on pH_45min_, drip loss, thawing loss, color at 45 min and 24 h, and meat texture (raw and cooked). Also, Al-Baadani et al. [26] reported that dietary gum Arabic (0.12% to 0.75% as a source of natural prebiotics) had a positive impact on pH_24h_, cooking loss, shear force, L* value, cohesiveness, resilience, gumminess, and chewiness, while pH_15min_ and WHC were not affected in the breast meat of broilers. These results suggest that dietary prebiotics may enhance the functional integrity of muscle proteins and connective tissue, leading to a uniform muscle structure and improving textural quality. Both Namted et al. [49] and Al-Baadani et al. [26] observed that cooking loss was reduced due to dietary prebiotic supplementation. From the biochemical standpoint, meat water retention is closely related to muscle protein functionality, pH, and muscle fiber organization and integrity. However, Yang et al. [50] reported that dietary inclusion of *Polygonatum sibiricum* polysaccharide (800 mg/kg) reduced the b* value of thigh meat; meanwhile, meat pH, drip loss, and cooking loss were not affected in breast and thigh muscles. Biswas et al. [48] reported that the addition of 0.2% mannan-oligosaccharides increased WHC and extract release volume, while pH and drip loss were significantly affected in broiler chickens. On the contrary, other studies pointed out that dietary prebiotics had no negative effect on key meat quality parameters [51,52,53]. Ruangpanit et al. [53] elucidated that 0.5% fructo-oligosaccharides supplementation had no negative impact on the physical characteristics of *Pectoralis* major muscle, involving pH, color, drip loss, cooking loss, and shear force. Yang et al. [52] pointed out that dietary inclusion of *Radix rehmanniae preparata* polysaccharide (300–900 mg/kg) had no adverse effect on color, pH_45min_, pH_24h_, drip loss, and cooking loss of the breast and thigh muscles. Nopparatmaitree et al. [51] reported that the addition of trimmed asparagus by-products (30 to 50 g/kg as a source of fructans, such as inulins and fructooligosaccharides) had no harmful effect on meat physical characteristics including pH_15min_, pH_24h_, color (L*, a*, and b* values), and WHC in the breast meat of broilers. It might be concluded that dietary prebiotics are effective functional feed additives that improve meat quality characteristics or at least do not have an adverse effect on breast and, in some studies, thigh muscle quality in broilers. Their main benefits are enhanced tenderness, improved texture profile parameters, reduced cooking loss, and reduced shear force, primarily reported in breast (*Pectoralis major*) muscle. It is noteworthy that two studies using a comparable fructo-oligosaccharide inclusion level (0.5% of diet) reported divergent physical-trait outcomes. Yan et al. [28] reported increased pH and b* value and decreased shear force, whereas Ruangpanit et al. [53] reported no significant effect on pH, color, drip loss, cooking loss, or shear force. This divergence may plausibly be related to differences in rearing environment between the two studies, since the broilers in Ruangpanit et al. [53] were specifically reared on reused litter, a management condition associated with elevated environmental and microbial challenge that may have altered the birds’ baseline physiological and gut-microbiota status relative to conventional rearing conditions.

Regarding the effect of dietary prebiotics on meat chemical composition, Wu et al. [27] reported that the addition of polysaccharides of *Morinda officinalis* (500 mg/kg) increased the concentration of total amino acids, total non-essential amino acids, and total PUFAs, whereas the addition of polysaccharides of *Morinda officinalis* (1000 mg/kg) significantly reduced the total SFA concentration in the breast meat of Wenchang chickens. Al-Baadani et al. [26] further reported that dietary gum Arabic (0.12% to 0.75% as a source of natural prebiotics) increased CP content, reduced crude fat content, and did not affect moisture, ash, and organic matter content in the breast meat of broiler chickens. They also noted that dietary gum Arabic (0.12%) increased n-3 PUFAs, n-6 PUFAs, and total PUFA concentrations in the breast meat, while concentrations of capric acid, lauric acid, palmitic acid, heptadecanoic acid, stearic acid, pristanic acid, and total SFAs were not affected. The increase in muscle CP and total amino acid content might be attributed to the fact that prebiotics may enhance amino acid availability and utilization via stabilizing gut microbiota and nutrient absorption. Nopparatmaitree et al. [51] noted that the addition of trimmed asparagus by-products (30 to 50 g/kg diet) reduced cholesterol, palmitic acid, oleic acid, SFAs, and MUFA concentrations in the breast meat of broilers, while n-3 PUFAs and n-6 PUFAs contents were not affected. Biswas et al. [48] observed that the addition of 0.2% mannan-oligosaccharides reduced cholesterol and fat content in broiler chickens. Interestingly, some studies indicated that dietary prebiotics had a favorable impact on the fatty acid profile by increasing PUFAs and reducing cholesterol and SFAs [26,27,51], which might indicate a beneficial modulation of hepatic lipid metabolism through the suppression, rather than enhancement, of de novo lipogenesis. Since de novo lipogenesis is the metabolic pathway responsible for the endogenous synthesis of SFAs from non-lipid precursors (primarily via acetyl-CoA carboxylase and fatty acid synthase activity), a reduction in SFAs and cholesterol concentrations following prebiotic supplementation more plausibly reflects downregulation of this pathway rather than its activation. This interpretation is consistent with the broader mechanistic evidence discussed in Section 3, where probiotic-induced changes in fatty acid profile were similarly linked to modulation of key lipogenic genes, including acetyl-CoA carboxylase alpha (ACCα), sterol regulatory element-binding protein-1c (SREBP-1c), and fatty acid synthase [23]; a parallel suppression of these lipogenic regulators by prebiotics would be expected to reduce SFA synthesis while sparing or favoring PUFA incorporation into muscle lipids. Taken together, it might be indicated that prebiotics could increase total amino acids, improve protein deposition, reduce fat content, and modify meat lipid profile toward enhancing the PUFAs concentration in breast muscle of broilers, offering benefits to human health.

**Figure 2:**
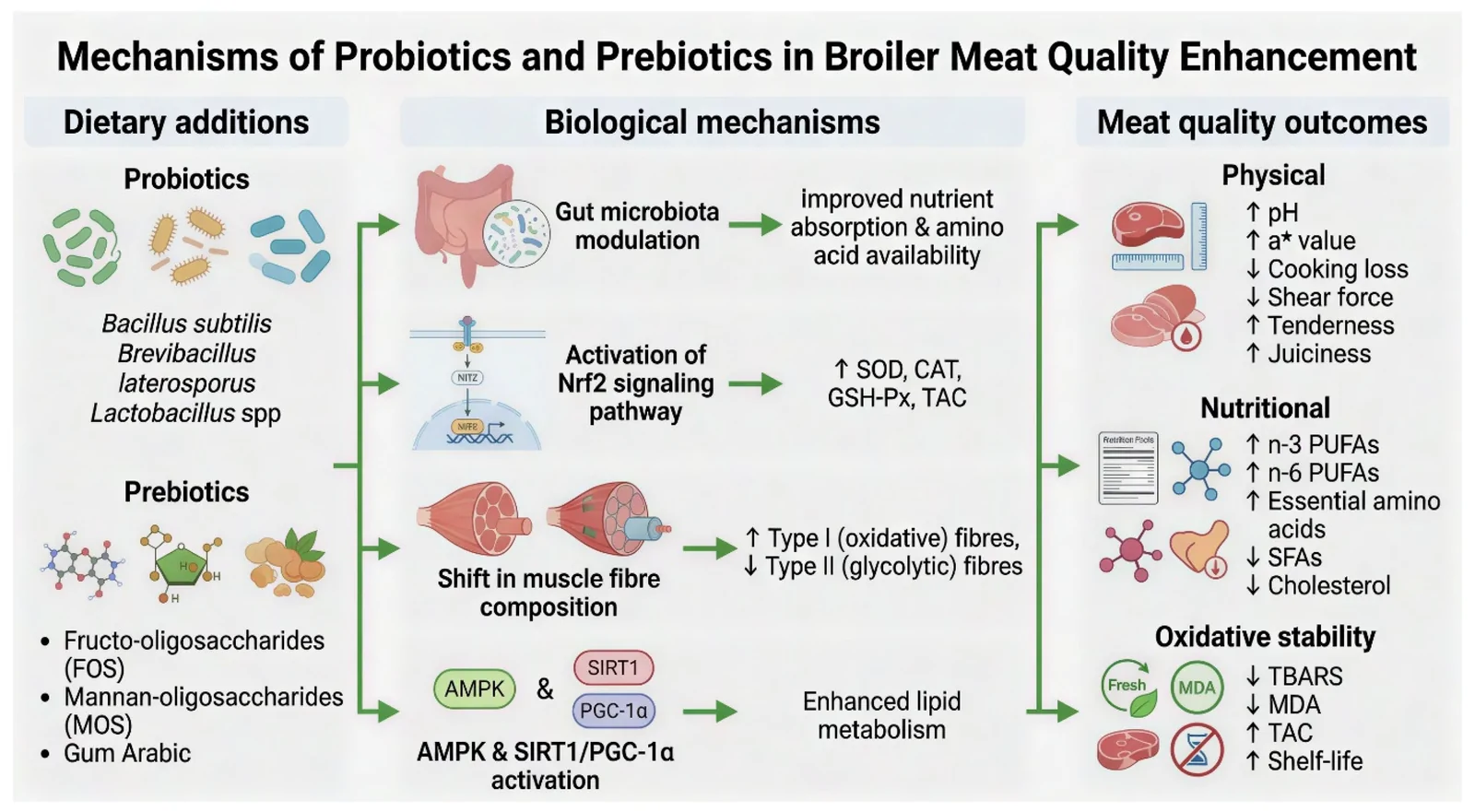
Conceptual diagram illustrating the mechanisms by which probiotics and prebiotics enhance broiler meat quality (This figure was created by the authors using Microsoft PowerPoint and BioRender.com).

It is interesting to mention that prebiotics could enhance antioxidant capacity and reduce meat lipid oxidation. Yan et al. [28] reported that dietary supplementation of 0.5% fructo-oligosaccharides increased TAC and decreased TBARS content in broiler breast and thigh meats. At the molecular level, they also postulated that gene expression of breast catalase (*CAT*), superoxide dismutase 1 (*SOD1*), and thioredoxin reductase 1 (*TXNRD*) were up-regulated by fructo-oligosaccharide addition. These enzymes play a vital role in scavenging free radicals and converting ROS to non-toxic molecules. Also, Huang et al. [54] observed that the addition of polysaccharides of *Morinda officinalis* minimized meat oxidative damage and, in turn, might be involved in the protection of muscle fibers from degradation, leading to improved meat quality and freshness. Moreover, Biswas et al. [48] elucidated that the addition of 0.2% mannan-oligosaccharides enhanced antioxidant and oxidative stability in the form of increasing meat concentrations of 1, 1-diphenyl-2-picrylhydrazy (an index of free radical scavenging activities) and reducing meat lipid oxidation indices (free fatty acids and TBARS) in broiler chickens. However, Ruangpanit et al. [53] demonstrated that 0.5% fructo-oligosaccharides supplementation had no significant effect on breast meat oxidative stability (TBARS values). Taken together, it might be mentioned that dietary prebiotic inclusion had a positive impact on meat oxidative stability in breast and thigh meats and reduced oxidative markers (TBARS/MDA) under the storage conditions examined, via enhancing antioxidant capacity, reducing meat lipid peroxidation, and improving gene expression of the key antioxidative enzymes—outcomes that reflect biochemical oxidative status rather than directly measured shelf-life endpoints (Figure 2).

## 5. Synbiotics

Synbiotics are a mixture of both probiotics and prebiotics in a single formulation, offering a synergistic effect that enhances the probiotics’ efficacy and survivability. True synergism is achieved only when the prebiotic substrate is selectively fermentable by the co-administered probiotic strain(s), generating a measurable downstream physiological cascade (e.g., SCFAs production, gut epithelial and immune modulation, and systemic antioxidant/metabolic effects) that is detectable across multiple, mechanistically connected endpoints rather than a single isolated trait [29,30,55]. Hamprakorn et al. [30] reported that dietary 0.1% synbiotics (*Lactobacillus johnsonii* and by-product of phycocyanin extraction probiotic) reduced pH_45min_ value and increased a* value in the breast meat of broiler chickens, while L* value, b* value, chroma, pH_24h_, WHC, drip loss, and shear force were not affected. Zhao et al. [56] observed that the addition of 0.1% synbiotics-enzyme complex (*Clostridium butyricum, Bacillus subtilis*, mannan-oligosaccharides, glucose oxidase, and α-galactosidase) reduced pH_24h_ and L* value and had no significant impact on a* value, b* value, or shear force of *Pectoralis* major muscle. This reduction in pH might reflect accelerated postmortem glycolysis. Moreover, Yalçın et al. [31] reported that dietary supplementation of 1 g/kg synbiotic (7 × 10^9^ CFU *Lactobacillus casei*, 7 × 10^9^ CFU *Lactobacillus acidophilus*, 7 × 10^9^ CFU *Bifidobacterium animalis* plus 40 mg inulin/chicory fiber) reduced shear force values, drip loss, and cooking loss, but increased pH_24h_ and WHC in the breast meat. In fact, Dev et al. [29] demonstrated that this response was dose-dependent on the prebiotic component: across a six-treatment design combining *L. acidophilus* (10^6^ or 10^7^ CFU/g) with graded mannan-oligosaccharide inclusion (0.1% or 0.2%), birds receiving the higher 0.2% mannan-oligosaccharide level consistently outperformed those receiving 0.1% mannan-oligosaccharide at the same probiotic dose, with breast and thigh meat pH decreasing progressively (breast: 5.89 to 5.82; *p* < 0.01) and WHC increasing correspondingly (breast: 81.6% to 87.5%; *p* < 0.05) as mannan-oligosaccharide inclusion rose. This provided direct evidence that increasing prebiotic substrate availability potentiates the efficacy of the same probiotic strain, the operational definition of synbiotic synergism. Complementary histomorphological evidence for the gut-level basis of these physical improvements comes from Pandey et al. [55], who reported that a multi-genus synbiotic (*Enterococcus*, *Bifidobacterium*, *Pediococcus*, and *Lactobacillus* spp. paired with chicory-root-derived fructo-oligosaccharides) significantly increased ileal villus height (1066–1080 µm vs. 1038 µm in controls; *p* < 0.01) and reduced crypt depth (128–129 µm vs. 133 µm; *p* < 0.05) in Cobb 500 broilers, consistent with an expanded absorptive epithelial surface area underlying the improved water-holding capacity reported across these studies. Notably, this structural benefit, together with significantly higher dressing percentage (69.6–72.4% vs. 60.9% in controls) and reduced cooking loss (22.5–23.8% vs. 29.1%) and drip loss (7.1–7.6% vs. 10.1%; all *p* < 0.05), was statistically similar regardless of whether synbiotic supplementation was provided for 7, 14, 21, or 28 days, indicating that these benefits are established during an early-life colonization window and do not require continuous supplementation to persist to market age. However, other studies indicated that dietary synbiotic had no detrimental effects on physical meat quality parameters [57,58]. Nopparatmaitree et al. [57] noted that the addition of synbiotic, consisting of 2 g/kg probiotics (*Lactobacillus acidophilus*, *Lactobacillus plantarum*, *Saccharomyces cerevisiae*, *Bacillus subtilis*, *Bacillus licheniformis*, *Streptococcus faecium*, and *Pediococcus pentosaceus*) plus 30 g/kg trimmed asparagus by-products (inulins and fructo-oligosaccharides) had no negative influence on meat color (L*, a*, and b* values), chroma, Hue angle, drip loss, cooking loss, thawing loss, and roasting loss in broilers’ meat. Tavaniello et al. [58] illustrated that synbiotic (*Lactobacillus reuteri*, *Enterococcus faecium*, *Bifidobacterium animalis*, *Pediococcus acidilactici,* and a fructo-oligosaccharide) administration neither in ovo (3 mg/embryo) nor in water (20 g/1000 birds/day) had any harmful effect on pH_24h_ and color (L*, a*, and b* values) of breast muscle. Collectively, the inclusion of synbiotics in broilers’ diet could influence pH levels, color intensity, and WHC in breast and, to a lesser extent, thigh muscle, though results often vary based on the specific microbial strains and oligosaccharides used. For example, Zhao et al. [56] found a synbiotic-enzyme complex (*Clostridium butyricum*, *Bacillus subtilis*, mannan-oligosaccharides, glucose oxidase, and α-galactosidase) decreased pH_24h_, while Yalçın et al. [31] found a different synbiotic blend (*Lactobacillus casei*, *Lactobacillus acidophilus*, *Bifidobacterium animalis*, plus inulin/chicory fiber) increased it at a higher dose. Different bacterial genera and the presence of added carbohydrate-active enzymes in Zhao et al. [56] formulation likely explain the divergence—the enzymes may have sped up postmortem glycolysis, an effect that does not present in Yalçın et al.’s enzyme-free blend. Further studies are still needed to determine the optimal dose and composition of synbiotics.

Regarding the nutritional quality of broiler meat, both Dev et al. [29] and Hamprakorn et al. [30] reported that dietary synbiotics improved CP content in the breast and thigh meat of chicken. Hamprakorn et al. [30] reported that 0.1% synbiotics (*Lactobacillus johnsonii* and a by-product of phycocyanin extraction probiotic) increased CP content, while not affecting gross energy, dry matter (DM), and crude fat contents in the breast meat of broilers. Dev et al. [29] proved that synbiotic supplements (1 × 10^6^ CFU/g *Lactobacillus acidophilus* and 0.2% mannan-oligosaccharides) increased protein level in the breast and thigh meat of chicken. These responses suggest that synbiotics may improve nitrogen utilization efficiency through improving amino acid absorption and gut integrity. Moreover, synbiotic supplementation is consistent with enhanced activation of the mechanistic target of rapamycin (mTOR) signaling pathway, the principal regulator of skeletal muscle protein synthesis [59].

From a lipid profile perspective, synbiotics improved the health-related lipid profile in broiler meat [57,60]. Nopparatmaitree et al. [57] posted that the addition of synbiotic consists of 2 g/kg probiotics (*Lactobacillus acidophilus*, *Lactobacillus plantarum*, *Saccharomyces cerevisiae*, *Bacillus subtilis*, *Bacillus licheniformis*, *Streptococcus faecium*, and *Pediococcus pentosaceus*) plus 30 g/kg trimmed asparagus by-products (inulins and fructo-oligosaccharides) reduced cholesterol, palmitic acid, oleic acid, palmitoleic acid, SFAs, MUFAs, and n-9 PUFAs, whereas Δ-9 desaturase (16) index and hypocholesterolemic/hypercholesterolemic ratio were significantly increased in chicken meat. Likewise, Dev et al. [60] reported that dietary supplementation with a synbiotic containing *Bifidobacterium bifidum* (1 × 10^6^ or 10^7^ CFU/g feed) and 0.2% mannan-oligosaccharides improved several meat health indicators in breast meat, including the PUFAs/SFAs ratio, MUFAs/SFAs ratio, hypocholesterolemic/hypercholesterolemic fatty acid ratio, saturation index, atherogenic index, thrombogenic index, and the content of hypercholesterolemic fatty acids. These authors also indicated that dietary synbiotic upregulated stearoyl CoA (∆9) desaturase-1 hepatic expression, which is involved in MUFA biosynthesis from corresponding SFAs. This gene-expression-level finding is corroborated compositionally by Dev et al. [29], who reported significantly elevated serum HDL-cholesterol alongside reduced total cholesterol and triglycerides in a dose-dependent manner across the same graded treatments. These results illustrate that a single upstream transcriptional and absorptive event can propagate into a coherent, multi-parameter improvement spanning both protein deposition and lipid quality simultaneously, which are considered a signature of true mechanistic synergism rather than additive, independent probiotic and prebiotic effects. However, Tavaniello et al. [58] showed that synbiotic (*Lactobacillus reuteri*, *Enterococcus faecium*, *Bifidobacterium animalis*, *Pediococcus acidilactici* and a fructo-oligosaccharide) administration, neither *in ovo* (3 mg/embryo) nor in water (20 g/1000 birds/day), had no significant effect on total lipids, cholesterol, and fatty acid profile (total SFA, PUFA, and MUFA contents) of Pectoralis major muscle. Overall, the findings suggested that synbiotics might serve as a powerful tool for enhancing meat healthiness via elevating protein contents and improving the fatty acid profile toward healthy fats (such as PUFAs and MUFAs) and lowering cholesterol content in breast meat and, in some studies, thigh meat.

Interestingly, several studies pointed out that dietary synbiotic supplementation improved the oxidative stability of broiler meat by reducing lipid oxidation and strengthening the antioxidant defenses [29,30,31]. Hamprakorn et al. [30] concluded that dietary 0.1% synbiotics (*Lactobacillus johnsonii* and a by-product of phycocyanin extraction probiotic) minimized TBARS concentrations on days 3 and 7 of storage in the breast meat of broilers. Also, Yalçın et al. [31] reported that dietary supplementation of 1 g/kg synbiotic (7 × 10^9^ CFU *Lactobacillus casei*, 7 × 10^9^ CFU *Lactobacillus acidophilus*, 7 × 10^9^ CFU *Bifidobacterium animalis* plus 40 mg inulin/chicory fiber) enhanced oxidative stability by reducing MDA concentration in the breast meat. Likewise, Dev et al. [29] demonstrated that synbiotic supplements (1 × 10^6^ CFU/g *Lactobacillus acidophilus* and 0.2% mannan-oligosaccharides) had a tendency to increase meat antioxidant capacity (2, 2-azinobis-3-ethylbenzothiazoline-6-sulfonic acid and 1, 1-diphenyl-2-picrylhydrazyl) and reduce concentrations of TBARS, peroxide, and free fatty acid in breast and thigh meat of chicken. This suggests that synbiotics not only reduce oxidative products but also strengthen the intrinsic ability of meat to neutralize free radicals.

## 6. Organic Acids

Regarding the impact of dietary organic acids supplementation on physical meat quality traits, some studies proved significant impacts [32,61,62,63,64], and others had no significant effects [7,8]. Gao et al. [32] reported that the addition of 1 g/kg of sodium butyrate reduced the L* value, cooking loss, and drip loss in the breast meat of broiler chickens. de-Cara et al. [62] proved that dietary 2 g/kg of protected sodium butyrate increased the a* value, Hue angle, and drip loss in the breast meat. Also, Akaichi et al. [63] showed that a dietary combination of humic acid (0.1%) and organic acids (0.02%) increased pH_24h_, WHC, and sensory evaluations, including color and smell of breast muscle. From a mechanistic point of view, improved WHC and reduced cooking loss and drip loss are likely associated with better protein functionality and reduced protein denaturation during postmortem glycolysis. This reflects a broader mechanistic principle applicable across additive classes: postmortem pH influences the net surface charge of myofibrillar proteins near their isoelectric point, thereby jointly governing WHC and L* value, so that pH, WHC, and color should be interpreted as interdependent rather than independent quality traits. This coupling is evident in several studies reviewed above and below (e.g., Section 3 and Section 5), where a lower ultimate pH consistently coincided with reduced WHC and increased lightness; notable exceptions, such as combined Se/Zn nanoparticle supplementation increasing both pH and L* value simultaneously, likely reflect additive-specific effects on protein solubility rather than a departure from this general rule. Ma et al. [61] observed that dietary 3000–6000 mg/kg of mixed organic acids (a blend of formic acid, ammonium formate, acetic acid, propionic acid, lactic acid, sorbic acid, and citric acid) elevated pH_24h_ and a* value, while drip loss, L* and b* values were not affected in breast and thigh muscles. On the other hand, several studies showed that dietary organic acids supplementation had no significant effects on physical meat quality traits [7,8]. Huerta et al. [8] reported that dietary sodium butyrate (150 or 300 mg/kg diet) had no harmful effect on meat physical characteristics, including pH, color (a*, b*, and L* values), thawing loss and cooking loss as well as sensory traits (cohesiveness, hardness, juiciness, chewiness, fibrosity, and flavor) in the pectoralis major muscle of broilers. Gu et al. [64] demonstrated that dietary coated sodium butyrate (250–1000 mg/kg) increased pH_24h_ and shear force, while having no negative effect on color (a*, b*, and L* values) of breast meat. Tellini et al. [7] reported that a dietary 150–300 g/t microencapsulated mixture of organic acids and bioactive compounds [calcium formate, citric acid, fumaric acid, orange essential oil (*Citrus sinensis*), thymol and carvacrol] had no negative impact on meat color (b* and L* values), thawing loss, cooking loss, and shear force in the breast meat of broilers. Collectively, the effect of organic acid supplementation on meat physical and sensory characteristics is not always consistent across breast (*Pectoralis* major) muscle, the tissue examined in nearly all cited studies, and this might be related to the differences between studies in dosage, the chemical form of acid, the composition of organic acids blend, sex, basal diet composition, and rearing environment. This area of research still needs further studies to clarify dose–response relationships and optimum organic acid formulations.

A substantial proportion of the inconsistency noted above is attributable to the physicochemical behavior of unprotected (uncoated) sodium/calcium butyrate specifically, as distinct from its protected, coated, or glyceride counterparts, and this distinction merits explicit mechanistic treatment given its centrality to interpreting the organic acids literature in broilers [65]. Unprotected butyric acid (pKa = 4.82) dissociates according to the local gastrointestinal pH, which ranges from approximately 4.5 in the crop to 2.5 in the gizzard; as long as ambient pH remains below the pKa of butyric acid, the acid remains predominantly undissociated and antimicrobially active, but upon reaching the more neutral, buffered environment of the duodenum, sodium or calcium salts rapidly release Na^+^/Ca^2+^ ions and the acid is largely neutralized before reaching the jejunum and ileum [65]. This site-restricted action explains why unprotected butyric acid/sodium butyrate exerts its primary physiological effects in the upper gastrointestinal tract (crop, proventriculus, gizzard, and duodenum) rather than throughout the entire intestine, in contrast to protected, fat-encapsulated, or glyceride forms, which are released further along the tract in a lipase-dependent manner and can therefore influence jejunal and ileal morphology and function more consistently [65]. This pharmacokinetic distinction provides a coherent explanation for why the meat-quality outcomes of unprotected butyric acid/sodium butyrate are reported in this review. For example, the significant improvements reported by Gao et al. [32] at a relatively high unprotected inclusion level (1 g/kg) contrasted with the null findings of Huerta et al. [8] at lower unprotected doses (150–300 mg/kg)—diverge according to dose and gastrointestinal exposure time rather than reflecting a fundamentally inconsistent mode of action.

Regarding meat nutritional value, dietary organic acid supplementation can positively modulate the lipid profile of broiler meat by balancing between PUFAs and SFAs [8,64,66]. Gao et al. [32] observed that the addition of 1 g/kg of sodium butyrate reduced free fatty acids, SFAs, myristic acid (C14:0), palmitic acid (C16:0), arachidonic acid (C20:4n6), and the n-6/n-3 PUFAs ratio, while PUFAs were increased in the breast meat of broiler chickens. Gu et al. [64] indicated that dietary coated sodium butyrate (250–1000 mg/kg) increased the meat content of PUFAs (palmitoleic acid, linoleic acid, docosatetraenoic acid, and docosahexaenoic acid), while having no effect on SFAs or MUFAs in the breast meat. Moreover, Ma et al. [61] elucidated that dietary 3000–6000 mg/kg of mixed organic acids (blend of formic acid, ammonium formate, acetic acid, propionic acid, lactic acid, sorbic acid, AL, and citric acid) reduced concentrations of palmitic acid and SFAs, while increasing concentrations of α-linolenic acid (C18:3 n3), linoleic acid (C18:2), PUFAs, and the PUFA/SFA ratio in the breast meat. Galli et al. [66] postulated that the addition of microencapsulated organic acids to the diet enhanced PUFA content and decreased total SFA content in broiler meat. From the physiological and metabolic perspective, these lipid profile changes might be linked to improved gut health, enhanced fat digestibility, absorption, and metabolism, including enzymes involved in fatty acid elongation and desaturation. This is consistent with the mechanism reported for unprotected butyric acid in turkeys, where supplementation with butyric acid glycerides was shown to enhance protein digestibility and reduce gastric pH, and where sodium butyrate has been demonstrated to lower gastric pH, increase conversion of pepsinogen to pepsin, and elevate cellular Ca^2+^ concentration in pancreatic islets, thereby increasing amylase secretion and, by extension, the digestive enzymatic capacity available for lipid as well as protein processing [65]. Unprotected butyric acid has additionally been shown to suppress bile-salt-degrading bacteria, which otherwise reduce nutrient utilization by microbial competition, providing a further gut-microbiota-mediated route by which unprotected butyric acid supplementation could support the improved fatty acid and nutrient profiles reported across the organic acid studies in this review [65]. Huerta et al. [8] showed that dietary sodium butyrate (150 or 300 mg/kg diet) had no negative effect on meat chemical composition, including moisture, CP, ash, and EE concentrations of Pectoralis major muscles of broilers. This indicates that organic acids did not disrupt the fundamental structural composition of muscle tissue. In conclusion, dietary organic acid supplementation had a positive impact on the fatty acid profile of breast muscle by increasing the ratio of essential fats, while maintaining the basic chemical composition of this muscle of broiler meat.

It is notable that dietary administration of organic acids reduced lipid oxidation and enhanced antioxidative status, biochemical outcomes that may contribute to, but do not directly establish, longer meat shelf life [7,32,62]. Tellini et al. [7] reported that the addition of 150–300 g/t microencapsulated mixture of organic acids and bioactive compounds [calcium formate, citric acid, fumaric acid, orange essential oil (*Citrus sinensis*), thymol and carvacrol] enhanced lipid oxidative stability via reducing lipid oxidation (MDA concentration) and enhancing antioxidant capability [2,2-difenil-1-picrilhidrazil (DPPH) and 2,2′-azino-bis (3-ethylbenzothiazoline-6-sulfonic acid); ABTS] in the breast meat of broilers. These results suggest a synergistic interaction between acidifiers and phytogenic antioxidants in stabilizing muscle lipid peroxidation. As a histone deacetylase (HDAC) inhibitor, butyrate can epigenetically upregulate transcription of endogenous antioxidant enzyme genes, providing a molecular route by which a gut-acting acidifier produces systemic, muscle-level antioxidant effects rather than acting merely as a local intestinal acidifying agent. This is consistent with Gao et al. [32], who reported that the addition of 1 g/kg of sodium butyrate enhanced the antioxidant capacity of meat (DPPH and ABTS concentrations) and minimized lipid oxidation (MDA concentration) in the breast muscle of broilers. This finding for unprotected sodium butyrate is directly reinforced by the NF-κB-suppression and antioxidant-enzyme-upregulation mechanism described above [65]. Unprotected butyric acid can suppress NF-κB-driven ROS generation and stimulate antioxidant gene expression even while acting predominantly in the upper gastrointestinal tract. Consequently, muscle-level antioxidant benefits can arise from a systemic reduction in host oxidative and inflammatory burden, rather than requiring direct butyric acid exposure to the muscle tissue itself. This is consistent with the muscle CAT activity increases and MDA reductions reported for sodium butyrate in this review, despite its restricted intestinal absorption site. de-Cara et al. [62] proved that dietary 2 g/kg of protected sodium butyrate reduced meat lipid oxidation (MDA concentration) at 0, 3, and 6 days of refrigerated storage of breast meat, a design that directly supports a shelf-life interpretation given its defined storage endpoint. This indicates that organic acids may indirectly modulate antioxidant status and lipid oxidation, leading to improved oxidative stability and, in studies employing a defined refrigerated-storage trial such as de-Cara et al. [62], a prolonged shelf life specifically in breast (*Pectoralis major*) meat, the muscle assessed in these studies. Beyond their direct antioxidant action, organic acids such as sodium butyrate are recognized modulators of inflammatory signaling. As a histone deacetylase (HDAC) inhibitor, butyrate suppresses nuclear factor kappa B (NF-κB) nuclear translocation and downstream transcription of pro-inflammatory cytokines (IL-1β, IL-6, TNF-α) in gut and, secondarily, systemic tissues [67].

## 7. Phytobiotics or Phytogenic Feed Additives

Phytobiotics, also known as phytogenic feed additives, are natural bioactive substances derived from plants, essential oils, and plant extracts that, either by themselves or in combination, enhance animal health, productivity, and product quality [68]. They have drawn interest due to their safety, distinct biological activities, non-toxicity, health benefits, and environmental sustainability. In recent years, considerable attention has been given to their potential to improve meat quality traits in broiler chickens, driven by the global movement to reduce the use of antibiotic growth promoters [33,34,69]. Ncho et al. [70] reported that dietary 0.5% or 1% polyphenolic extract had no negative effect on color values (L*, a*, and b*), chroma, Hue angle, cooking loss, and shear force, while the addition of 0.5% polyphenolic extract reduced pH values of breast muscle. Ren et al. [71] noted that dietary provision of 0.8% phytogenic feed additive (mixture of Radix *Codonopsis pilosulae, Radix Astragali*, *Poria*, *Rhizoma Atractylodis macrocephalae*, *Fructus Hordei germinatus Preparata*, *Massa Medicata Fermentata*, *Crataegus pinnatifida*, and *Alpinia katsumadai*) reduced pH_1h_ and increased a* value, whilst L* and b* values, cooking loss and pressurized water loss rate did not significantly affected on white-feathered broilers. Both Al-Garadi et al. [34] and Kahiel et al. [43] documented that the addition of phytogenic feed additives (wheat straw and sweet-cane hydrolyzed fiber, glycerol, lactic acid, citric acid, and benzoic acid) or essential oil blends (extractions of oregano leaves, cinnamon peels, and orange peels) improved WHC and reduced drip loss and cooking loss of breast meat. These effects might be attributed to the antioxidant properties of phytochemicals, which limit protein oxidation and preserve muscle structural integrity. Hossain et al. [72] proved that the addition of 0.05% herbal mixture (mixture of ginseng and artichoke) to broiler diets improved a* value and WHC in breast meat, while pH, cooking loss, drip loss, L* value, and b* value were not changed significantly in the breast meat. Obeidat et al. [69] showed that dietary provision of 150 g/ton feed essential oils (oregano, caraway, carvacrol, methyl salicylate, and menthol) reduced cooking loss; meanwhile, pH, WHC, and Warner-Bratzler shear force were not affected in the breast meat. Michalczuk et al. [33] postulated that 100 ppm of a phytogenic additive (turmeric, red pepper, white mustard, sweet flag, and soapwort) improved WHC, increased a* value, reduced drip loss, and reduced cutting force value; however, pH_24h_, thermal loss, L* value, and b* value were not significantly affected in breast muscles. The phytogenic additive-group meat showed a more favorable volatile organic compounds profile (higher aroma units, including amines, ketones, and aldehydes). These results suggest that phytobiotics not only affect physical quality parameters but also enhance flavor precursors and aroma-active compounds. This finding is noteworthy because it suggests that the relationship between oxidative stability and sensory acceptance may follow a threshold rather than a linear pattern. Certain oxidation-derived aldehydes contribute, at moderate concentrations, to desirable “meaty” flavor notes, so a dose-appropriate antioxidant effect may enhance rather than diminish palatability. However, excessive suppression of oxidation could theoretically blunt these flavor precursors. Few of the reviewed studies, however, concurrently assessed antioxidant dose, oxidative markers, and sensory panel scores, underscoring the need for future dose–response trials that define the inclusion levels at which further antioxidant enhancement ceases to benefit, or may even compromise, consumer-perceived meat quality. Conversely, other studies [73,74] reported no significant effect on fundamental meat physical quality traits in broilers. Mullenix et al. [74] concluded that 75–100 g/ton of phyto/phycogenic mixture (encapsulated micro-particles of dried kelp, cinnamaldehyde, thymol, and eugenol) did not affect pH_24h_, L* value, a* value, drip loss, and sensory traits (aroma, flavor, and taste/texture) in breast muscle. Iwiński et al. [73] pointed out that the addition of 1–2 mL/L phytobiotic mixture (monoterpenoid, phenylpropanoid, aromatic natural compounds—*trans*-anethole, 1,8-cineole, menthol) to drinking water had no significant impact on pH_15min_, pH_60min_, pH_24h_, color (a*, b*, and L* values), WHC, tenderness, and natural leakage in breast and thigh meats. In conclusion, administration of phytobiotics or phytogenic feed additives could improve the WHC, tenderness, and color of broiler breast meat specifically, since the majority of the reviewed studies assessed the Pectoralis major muscle; evidence from thigh or leg muscle remains comparatively limited. Some herbal blends or extracts had a negligible impact on meat physical properties in breast muscle.

Considering meat nutritional value, several studies indicated that dietary phytobiotics did not alter the proximate composition of broiler meat [33,70,75]. Michalczuk et al. [33] postulated that 100 ppm of a phytogenic additive (turmeric, red pepper, white mustard, sweet flag, and soapwort) had no significant impact on meat chemical composition (moisture, EE, and CP), while collagen content was increased in breast muscles. Ncho et al. [70] reported that dietary 0.5% or 1% polyphenolic extract had no harmful effect on proximate composition (moisture, CP, EE, and ash) of breast muscle. Bonos et al. [75] elucidated that a herbal mixture of oregano, garlic, sage, and rock samphire extracts (3 g/kg feed) had no negative effect on chemical composition (moisture, CP, EE, and ash) in the breast and thigh meat of broilers. These findings suggest that phytobiotics do not interfere with protein accretion or muscle development. Interestingly, dietary herbs and their bioactive constituents enhanced meat nutritional value by changing the fatty acid profile [76]. Recently, Kahiel et al. [43] demonstrated that supplementation with essential oil blends (extractions of oregano leaves, cinnamon peels, and orange peels) in the drinking water (250 mg/L) increased MUFAs and PUFAs, while SFAs were decreased in the breast meat. In summary, the basic chemical composition of breast meat was unaffected by the addition of phytobiotics; however, they enhanced the fatty acid profile of breast muscle by increasing the healthy fatty acids such as MUFAs and PUFAs.

**Figure 3:**
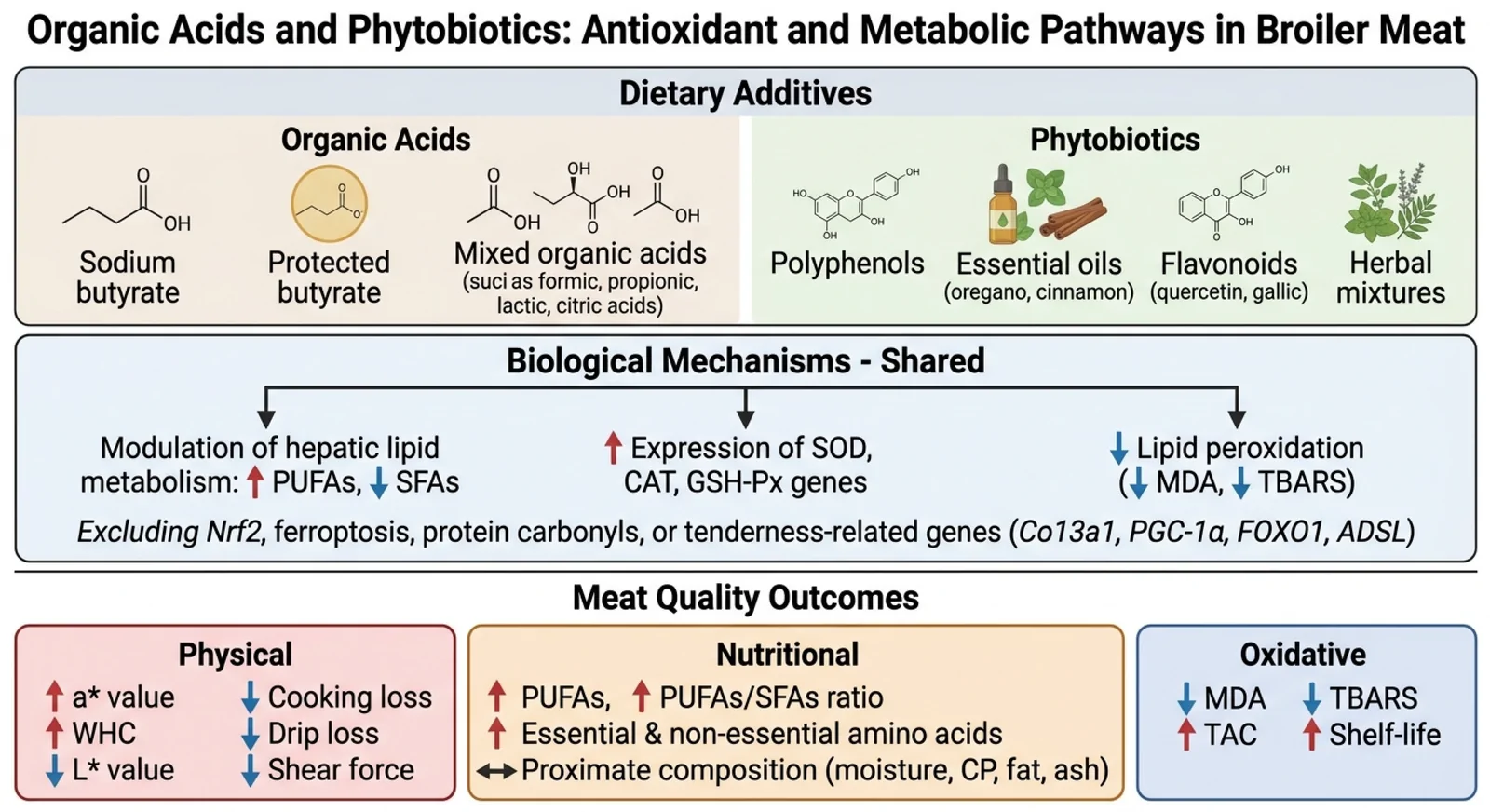
Conceptual diagram showing the antioxidant and metabolic pathways of organic acids and phytobiotics in broiler meat (This figure was created by the authors using Microsoft PowerPoint and BioRender.com).

Phytobiotics or phytogenic feed additives possess antioxidants that can strengthen the antioxidant system and suppress lipid oxidation in broiler meat. Ncho et al. [70] reported that dietary 1% polyphenolic extract reduced lipid oxidation marker (TBARS) values in the breast meat. Kahiel et al. [43] demonstrated that essential oil blend (extractions of oregano leaves, cinnamon peels, and orange peels) supplementation in the drinking water (250 mg/L) improved the antioxidative properties (increased GSH-Px, CAT, and TAC concentrations and reduced MDA content) and expression of antioxidant-related genes (mRNA levels for *SOD*, *CAT*, and *GSH-Px*) in muscles, as well as gene expression of liver FA synthesis, which was downregulated, while the expression of lipid β-oxidation-related genes was elevated in the breast meat. Also, Bonos et al. [75] elucidated that a herbal combination of oregano, garlic, sage, and rock samphire extracts (3 g/kg feed) minimized lipid oxidation (MDA concentrations) in both breast and thigh meat of broilers. These effects might be attributed to the bioactive constituents of phytobiotics such as alkaloids, glycosides, phenols, and terpenoids, which improve the antioxidative properties. Generally, phytogenic feed additives offer a broad potential for improving meat quality, primarily demonstrated in breast (*Pectoralis* major) muscle in poultry, but their effects vary depending on the type, ratio, and dosage used. Therefore, more detailed research is required to identify the most effective and safe combinations (Figure 3).

## 8. Trace Minerals

Currently, there is a great interest in using organic minerals and nanoparticles in animal and poultry nutrition. Nano-minerals have garnered increased interest due to their high surface activity, great bioavailability, strong catalytic effectiveness, excellent absorption capacity, and lower toxicity [77]. Also, organic minerals are characterized by enhanced stability, absorption, and biological availability compared with conventional inorganic mineral salts [78]. Studies elucidated that organic Se, Zn, and Mn [4,35,79] or nanoparticles [36,80] had a significant impact on meat quality characteristics. Nie et al. [35] reported that dietary of 300–500 mg/kg of amino acid-chelated organic trace minerals premix including 1.8 × 10^5^ mg/kg Fe, 1.8 × 10^5^ mg/kg Cu, 1.6 × 10^5^ mg/kg Zn, and 1.6 × 10^5^ mg/kg Mn increased pH_45min_, reduced drip loss_24h_, and reduced drip loss_48h_, meanwhile meat color indices (a*, b*, and L*), cooking loss, and shear force were not affected in the breast muscle of Chinese yellow-feathered broilers. The elemental concentrations reported for this premix (1.8 × 10^5^ mg/kg Fe, 1.8 × 10^5^ mg/kg Cu, 1.6 × 10^5^ mg/kg Zn, and 1.6 × 10^5^ mg/kg Mn) represent premix-basis values, i.e., the elemental content per kilogram of the concentrated premix itself, and not the final elemental concentration in the complete diet. At the stated inclusion rate of 300–500 mg premix/kg diet, these premix-basis values correspond to final dietary concentrations of approximately 54–90 mg Fe/kg and 54–90 mg Cu/kg complete feed, and approximately 48–80 mg Zn/kg and 48–80 mg Mn/kg complete feed. Notably, the resulting copper concentration exceeds the EU regulatory maximum permitted level for complete poultry feed (25 mg Cu/kg complete feed for chickens for fattening; Regulation (EU) 2018/1039) by a factor of approximately 2.2 to 3.6, and the calculated Fe, Zn, and Mn concentrations likewise fall well above concentrations typically reported for commercial trace-mineral premix supplementation in broiler diets. This discrepancy may reflect a transcription or unit-reporting error in the original source (e.g., a possible one-order-of-magnitude overstatement of premix-basis elemental concentrations, or a different intended inclusion-rate unit), rather than values genuinely intended for practical diet formulation at these levels. Readers and practitioners should therefore treat the elemental concentrations and resulting calculations reported here as derived strictly from the values stated in Nie et al. [35] and should consult the original publication directlyto confirm the intended premix-basis concentrations and resulting dietary elemental intake before applying these figures to practical diet formulation or drawing regulatory-compliance conclusions. From another point of view, it is worth noting that Chinese yellow-feathered broilers are a slow- to medium-growing genotype characterized by a lower growth rate, greater proportion of oxidative muscle fibers, and generally higher meat quality attributes (e.g., lower drip loss, more desirable color) compared with fast-growing commercial broiler hybrids; therefore, the magnitude of the mineral-mediated improvements reported in this genotype may not be directly comparable to, or fully replicable in, fast-growing broiler lines with a distinct muscle fiber profile and postmortem metabolic rate. However, Konkol et al. [79] showed that dietary Se-enriched yeast (0.1 g/1 kg diet) had no significant effect on pH and WHC in breast and thigh muscles of broilers. This indicates that these responses may depend on mineral form, level, and baseline dietary adequacy. Several studies mentioned that dietary Se nanoparticles (SeNPs) had an impact on meat quality characteristics [36,80]. Huang et al. [36] proved that dietary 5 mg/kg biogenic SeNPs synthesized with alginate oligosaccharides decreased b* value, reduced freezing loss, increased pH_0min_, and had no significant impact on pH_15min_, pH_24h_, L* value, a* value, and the shear force of thigh muscle in broiler chickens under heat stress conditions. These results suggest that SeNPs had a protective role against stress-induced muscle deterioration. Alhasani [80] noted that dietary nanoparticles of 1.5 mg/kg SeNPs, 2.0 mg/kg ZnNPs, or a combination of 1.5 mg/kg SeNPs + 2.0 mg/kg ZnNPs increased pH, L* value, and b* value and reduced tenderness of the major pectoral muscle in broiler chickens. These results indicated that antioxidant benefit and textural quality do not necessarily co-vary and may represent a trade-off rather than a uniformly positive outcome. Moreover, this finding illustrates that improvements in color indices do not necessarily translate into improved tenderness, as these traits are governed by distinct biological mechanisms. Color primarily reflects myoglobin chemistry and light-scattering properties, whereas tenderness reflects connective tissue content and postmortem proteolytic activity (e.g., the calpain system). Consequently, a favorable color response to a given additive should not be assumed to indicate a proportional tenderness benefit unless confirmed by direct shear-force or myofibrillar fragmentation measurements. Likewise, Abd El-Hack et al. [81] postulated that dietary provision of 0.2 and 0.4 mg ZnNPs/kg diet increased pH_24h_, L* value, and b* value, while sensory properties (aroma, taste, tenderness, and juiciness) were not affected in the main pectoral muscle. Onbaşılar et al. [4] elucidated that Zn amino acid complex supplementation (50 mg/kg) reduced the incidence of spaghetti meat and wooden meat in the breast meat. These findings suggest that trace minerals may play a role in maintaining muscle structural integrity via their antioxidative properties, as they are cofactors in antioxidative enzymes (e.g., Se in GSH-Px and Zn in SOD). However, Winiarska-Mieczan et al. [82] demonstrated that dietary 56.92 or 98.63 mg Zn glycine chelate/kg diet did not affect pH_15min_ and pH_45min_ in thigh meat. Li et al. [44] concluded that Mn amino acid complex (40, 80, 120, or 160 mg Mn/kg) increased a* value, pH_45 min_, and pH_24 h_, reduced shear force, drip loss, and pressure loss of breast muscle; however, b* and L* values were not affected. Nadaf Fahmideh et al. [83] reported that dietary administration of 700 mg organic Cr/kg diet increased fat content, tenderness, odor, oral sensation, and general acceptance, while color, juiciness, chewing ability, and elasticity were not affected. In conclusion, incorporating trace elements like Se, Zn, and Mn in the form of organic minerals or nanoparticles improved physical meat characteristics such as color, drip loss, freezing loss, and shear force in breast and thigh muscles, as reported across the cited studies, as well as reduced the severity of breast meat myopathy (specifically in Pectoralis major muscle). Further studies are required to clarify molecular mechanisms involved in muscle metabolism and development.

Regarding meat chemical composition, Alhasani [80] noted that dietary nanoparticles of 1.5 mg/kg SeNPs, 2.0 mg/kg ZnNPs, or a combination of 1.5 mg/kg SeNPs + 2.0 mg/kg ZnNPs increased moisture content and decreased amounts of fat, ash, and total volatile basic nitrogen of major pectoral muscle in broiler chickens. Also, Giamouri et al. [45] pointed out that dietary addition of SeNPs (0.4 mg/kg) increased concentrations of docosahexaenoic acid (22:6 n−3), total n−3 PUFAs, and the n−3/n−6 ratio in the breast meat of broiler chickens; however, total SFAs, total MUFAs, and total PUFAs were not affected. Onbaşılar et al. [4] elucidated that Zn amino acid complex supplementation (50 mg/kg) had a positive effect on DM and CP content in the breast meat; however, ash content, fat content, fatty acid profile, and free amino acid composition were not affected in the breast meat. Winiarska-Mieczan et al. [82] demonstrated that dietary 56.92 or 98.63 mg Zn glycine chelate/kg diet did not affect chemical composition (moisture, EE, CP, and total cholesterol) in thigh meat; however, fatty acid profile was significantly affected in the form of increasing total content of n−3 PUFAs, PUFA/SFA ratio, and n-3/n-6 ratio; however, total content of SFAs, MUFAs, and n−6 PUFAs were not affected in thigh meat. Abd El-Hack et al. [81] postulated that dietary supplementation of 0.2 and 0.4 mg ZnNPs/kg diet increased moisture, CP, and reduced EE, ash, and total volatile basic nitrogen in the main pectoral muscle. It might be concluded that adding Se and Zn nanoparticles can effectively reduce fat content while increasing moisture, protein, and healthy n-3 PUFA concentrations in breast (major pectoral) muscle.

**Figure 4:**
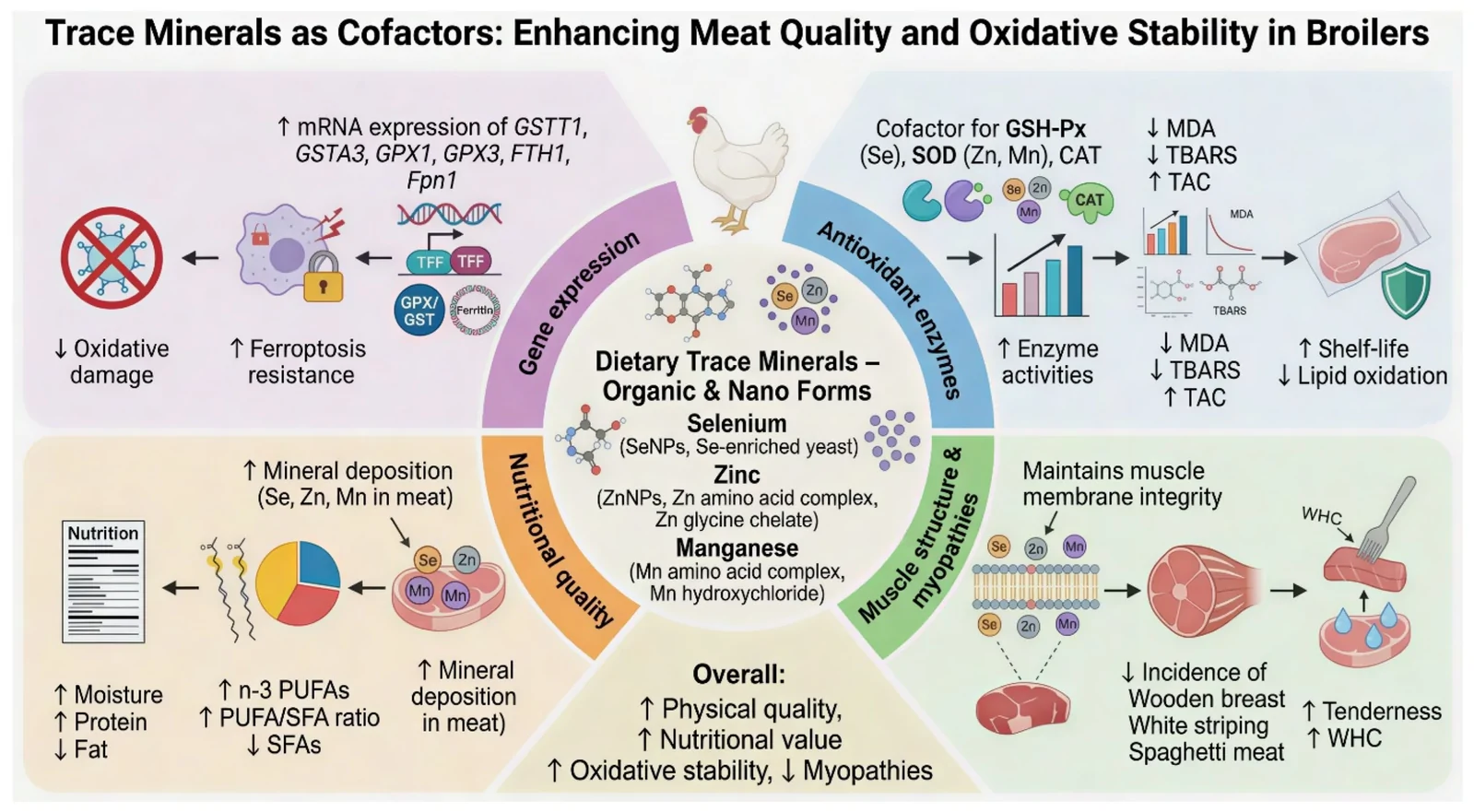
A radial conceptual diagram illustrates the roles of organic and nano forms of selenium, zinc, and manganese in improving broiler meat quality (This figure was created by the authors using Microsoft PowerPoint and BioRender.com).

Nie et al. [35] stated that a dietary of 300–500 mg/kg of amino acid-chelated organic trace minerals premix including 1.8 × 10^5^ mg/kg Fe, 1.8 × 10^5^ mg/kg Cu, 1.6 × 10^5^ mg/kg Zn, and 1.6 × 10^5^ mg/kg Mn increased meat Zn, Mn, Fe and Cu concentrations as well as enhanced CAT activity in the breast muscle of Chinese yellow-feathered broilers. These results are in harmony with Konkol et al. [79], who showed that dietary Se-enriched yeast (0.1 g/kg diet) increased Se concentration and reduced TBARS content in the breast and thigh muscles of broilers. Similarly, Alhasani [80] noted that dietary nanoparticles of 1.5 mg/kg SeNPs, 2.0 mg/kg ZnNPs, or a combination of 1.5 mg/kg SeNPs + 2.0 mg/kg ZnNPs decreased TBARS concentrations in the major pectoral muscle of broiler chickens. Giamouri et al. [45] pointed out that dietary addition of SeNPs (0.4 mg/kg) increased Se concentration and enhanced oxidative stability by reducing MDA levels in the breast meat of broiler chickens. Huang et al. [36] proved that dietary 5 mg/kg biogenic SeNPs synthesized with alginate oligosaccharides enhanced the activities of SOD, GSH-Px, glutathione S-transferase (GST), and improved the mRNA expression levels of *GSTT1*, *GSTA3*, *GPX1*, *GPX3*, ferritin heavy polypeptide-1 (*FTH1*), and ferroportin 1 (*Fpn1*) of thigh muscle in broiler chickens under heat stress conditions. Onbaşılar et al. [4] elucidated that Zn amino acid complex supplementation (50 mg/kg) enhanced TAC and reduced the oxidative stress index, while DPPH radical scavenging activity and total phenolic content were not affected in the breast meat. Also, Winiarska-Mieczan et al. [82] demonstrated that dietary 56.92 or 98.63 mg Zn glycine chelate/kg diet increased Zn concentration in thigh meat in a dose-dependent manner. Abd El-Hack et al. [81] postulated that dietary inclusion of 0.2 and 0.4 mg ZnNPs/kg diet decreased TBARS in the main pectoral muscle. Li et al. [44] concluded that Mn amino acid complex (40, 80, 120, or 160 mg Mn/kg) increased meat Mn content, increased GSH-Px and SOD activities, increased *SOD* gene expression, and reduced *MuRF1* and Atrogin-1 mRNA levels in the breast muscle. Similarly, Sun et al. [84] indicated that dietary 50–90 mg/kg of Mn hydroxychloride had a positive impact on SOD, CAT, GSH-Px, and TAC concentrations and minimized MDA concentrations in the breast and thigh muscles of broilers. Mechanistically, trace minerals play a vital role as essential cofactors in antioxidant enzymes (GSH-Px, SOD, and CAT), leading to reduced oxidative degradation of lipids and preserving PUFAs. Recently, Hamid et al. [85] provided direct dose-responsive gene-expression evidence for this bioavailability advantage in broilers: dietary nano-selenium (0.3–0.9 mg/kg) produced a dose-dependent upregulation of hepatic *Nrf2* (up to 3.80-fold) and its downstream targets *HO-1* (4.20-fold), *GSH-Px1* (3.45-fold), *SOD1* (3.15-fold), and *CAT* (2.95-fold) relative to control, significantly exceeding the transcriptional response achieved with an equivalent dose of organic selenomethionine, alongside parallel increases in serum GSH-Px, SOD, and total antioxidant capacity and a corresponding decline in MDA. It might be mentioned that dietary trace minerals in the form of organic and nanoparticles increased their concentrations in the breast and thigh muscles specifically, which consequently had a positive impact on oxidative stability, as reflected by reduced TBARS/MDA and enhanced antioxidant enzyme activity (which are biochemical outcomes consistent with, but not a direct substitute for, measured shelf-life endpoints). These trace elements activate the antioxidative enzymes by supplying rate-limiting catalytic cofactors (Se for GSH-Px, Zn/Cu/Mn for SOD, Fe for CAT) that directly determine enzyme Vmax, while simultaneously regulating their gene expression in muscles through MTF-1-mediated (zinc-specific) and Keap1–Nrf2–ARE-mediated (redox-responsive, broadly shared) transcriptional pathways, together explaining the coordinated activity–expression responses reported across selenium, zinc, and manganese sources in this section (Figure 4).

## 9. Vitamins

Vitamins are essential organic compounds in poultry diets and play a key role in improving meat quality attributes. Hassan et al. [37] proved that dietary inclusion of 150 mg vitamin E/kg diet increased WHC, plasticity, color (a*, b*, and L* values), and juiciness, but cooking loss, drip loss, thermal shortening, and cold shortening were decreased in the breast meat. Similarly, De Grande et al. [86] noted that dietary supplementation of vitamin E (50 or 100 IU vitamin E/kg diet) alone or in combination with 60 mg organic Zn/kg reduced drip loss and thawing loss in the breast meat; however, color indices (L*, a*, and b* values) were not affected. These results indicated that dietary inclusion of vitamin E had a positive effect on meat physical quality parameters. The beneficial effect of vitamin E on WHC and drip loss may be related to its ability to protect cellular and sarcolemmal membranes from oxidative damage, thereby preserving membrane integrity and limiting the release of intracellular water during post-mortem storage. Regarding vitamin C, Nadaf Fahmideh et al. [83] stated that the dietary addition of 250 mg vitamin C/kg diet increased fat content, tenderness, odor, oral sensation, and general acceptance, but reduced chewing ability and elasticity, while color and juiciness were not affected. These results illustrated that a single additive can simultaneously improve some sensory dimensions while impairing others. Also, Yu et al. [87] elucidated that the addition of 200 mg/kg vitamin C had no adverse effect on pH_1h_, pH_24h_, WHC, and color (L*, a*, and b* values) in the breast meat. As a water-soluble antioxidant, vitamin C primarily contributes to the aqueous antioxidant environment and can support the regeneration of lipid-phase antioxidants such as vitamin E; however, its direct effects on poultry meat water-holding capacity and oxidative stability appear to be dependent on dose, dietary composition, and the oxidative challenge. Moreover, Pečjak et al. [46] reported that dietary inclusion of vitamin E (200 IU vitamin E/kg diet) alone or in mixture with vitamin C (250 mg vitamin C/kg diet) and organic Se (0.2 mg Se/kg diet) had no significant influence on pH_15min_, pH_24h_, drip loss, and electrical conductivity of breast meat. The absence of significant changes in these physical traits despite improved antioxidant status emphasizes that oxidative protection and water-holding capacity are related but distinct components of post-mortem meat quality. Concerning vitamin A, Savaris et al. [88] demonstrated that dietary supplementation of vitamin A (6000, 16,000, 26,000, 36,000, and 46,000 IU/kg) enhanced b* value, but had no significant impact on pH_24h_, shear force, cooking loss, WHC, and MDA concentration in Pectoralis major muscle of broiler chickens. Wang et al. [38] postulated that dietary inclusion of vitamin A (5000 IU/kg) reduced drip loss, b* value and L* value, and increased pH_45min_, pH_24h_ and shear force of the breast muscle. A higher shear force value directly indicates greater mechanical resistance of the myofibrillar and connective tissue matrix, which is perceived by the consumer as tougher, chewier meat. It is noteworthy to indicate that vitamin A is a lipid-soluble vitamin and participates in maintaining cellular differentiation and membrane-associated biological functions; nevertheless, the present evidence indicates that its effects on post-mortem water retention and oxidative stability are less consistent than those reported for vitamin E. In particular, the lack of an effect of vitamin A supplementation on MDA concentration reported by Savaris et al. [88] suggests that increased dietary vitamin A should not be considered equivalent to vitamin E supplementation with respect to protection against post-mortem lipid oxidation. Wei et al. [39] concluded that dietary inclusion of 3600 IU/kg of vitamin D3 decreased the b* value of breast meat but had no significant impact on the L* value, a* value, pH_45min_, pH_24h_, cooking loss, and drip loss in the breast and thigh muscles. Similarly, Gao et al. [32] pointed out that dietary administration of vitamin D3 (5000 IU/kg diet) reduced a* value and cooking loss; however, L* value, b* value, pH_45min_, pH_24h_, drip loss, and shear force were not affected in the breast meat. Collectively, the impact of dietary vitamins on meat physical quality in breast muscle is variable and, in several cases, directionally inconsistent even within the same vitamin: vitamin A supplementation, for example, was reported to increase b* value in Savaris et al. [88] but decrease b* value in Wang et al. [38] at comparable inclusion levels, illustrating that its effects on meat color are not merely absent but can be directionally opposite between studies. Vitamins E and C most consistently produced favorable effects on WHC, drip loss, and related traits across the cited studies, whereas vitamins A and D3 should be more accurately characterized as producing inconsistent or context-dependent effects on physical meat quality traits, rather than uniformly “no negative impacts,” given the directional disagreement documented above. This area of research still needs further studies to clarify optimal inclusion levels and interactions with different nutritional and production conditions.

Regarding proximate composition and oxidative stability of meat, Pečjak et al. [46] reported that dietary supplementation of vitamin E (200 IU vitamin E/kg diet) alone or in mixture with vitamin C (250 mg vitamin C/kg diet) and organic Se (0.2 mg Se/kg diet) significantly raised the concentrations of α-tocopherol and Se, decreased MDA content, and had no significant influence on fatty acid profile of breast meat. Hassan et al. [37] proved that dietary inclusion of 150 mg vitamin E/kg diet increased moisture content, reduced EE content, and had no significant effect on CP and ash contents of breast meat. Nadaf Fahmideh et al. [83] revealed that dietary supplementation of 250 mg vitamin C/kg diet elevated fat content in the breast meat. Yu et al. [87] elucidated that the addition of 200 mg/kg vitamin C had no negative impact on TBARS in the breast meat. Gao et al. [32] pointed out that dietary supplementation of vitamin D3 (5000 IU/kg diet) reduced MDA in the breast meat, while meat chemical composition (DM, CP, EE, and ash) and meat lipid profile (SFAs, MUFAs, PUFAs, n−3PUFAs, n−6PUFAs, PUFA/SFA ratio, and n-3/n-6 ratio) were not affected. Although vitamin D3 is not conventionally regarded as a direct chain-breaking antioxidant comparable to vitamin E, its potential contribution to muscle homeostasis and oxidative status may influence post-mortem meat stability indirectly through regulation of cellular metabolism and antioxidant defenses. Importantly, Gao et al. [32] observed a reduction in MDA concentration following vitamin D3 supplementation, providing evidence that vitamin D3 may contribute to attenuation of lipid peroxidation even when its effects on physical meat quality traits are limited. It might conclude that vitamins E and D3 are effective in enhancing oxidative stability by lowering the lipid peroxidation index in breast meat, but they did not have a fundamental effect on basic chemical composition or fatty acid profile.

## 10. Natural Antioxidants

Natural antioxidants offer several advantages over synthetic vitamins as they have strong antioxidative properties, greater safety, lower risk of toxicity at high inclusion levels, and consumer preference. Studies confirmed that vitamin E could be partially substituted with natural antioxidants without adverse impacts on meat quality characteristics [47,89]. Andreaki et al. [89] indicated that partial substitution of synthetic vitamin E (50%) with 50 mg polyphenols as vinification by-products/kg feed from grape pomace extract decreased cooking loss, while having no effect on pH_24h_, shear force, and color (a*, b*, and L* values) in the breast muscle. Also, Corrales et al. [47] pointed out that substitution of 40 mg vitamin E/kg with 22.5 mg olive oil by-product extract rich in hydroxytyrosol/kg + 10 mg vitamin E/kg had no adverse effect on pH, color (a*, b*, and L* values), and drip loss in the breast muscle. In addition, Ahsan et al. [90] postulated that dietary supplementation of 60 mg/kg natural antioxidants blend (capsaicin, glucosinolates, saponins, terpenes, and curcumin) had no significant effect on pH_24h_, color (a*, b*, and L* values), drip loss, and cooking loss in the breast muscle. Cao et al. [91] elucidated that dietary bamboo leaf flavonoids (50 or 250 mg/kg diet) increased elasticity of breast meat, improved the gumminess of breast and leg muscles, lessened hardness of breast muscle, enhanced a* value and chroma in the breast muscle, and elevated chroma and WHC in leg muscle. Similarly, Hu et al. [92] demonstrated that dietary natural flavonoid quercetagetin (10 mg/kg) improved a* value, decreased cooking loss, and had no significant effect on pH_45min_, pH_24h_, L* value, b* value, shear force, and drip loss in breast meat. Regarding carotenoids, Yang et al. [40] concluded that dietary 200 mg/kg lycopene (natural red open-chain hydrocarbon carotenoids) increased L* value, a* value, elasticity, gumminess, and resilience, but reduced pH and b* value, while having no significant effect on hardness, chewiness, and cohesiveness in the breast meat. Also, Wu et al. [93] pointed out that the addition of 30 mg/kg dietary lycopene to the broilers’ feed had no negative effect on meat physical traits (pH_45min_, pH_24h_, L* value, b* value, and cooking loss) in both breast and leg muscle, while drip loss was reduced in the breast meat and shear force was reduced in leg meat. Regarding polyphenolic compounds, Serra et al. [94] reported that dietary 2 g/kg polyphenols (Oxilem^®^ or OxiGem^®^) increased a* value, reduced Hue angle, and had no significant effect on pH, L* value, b* value, and chroma in the breast muscle. Likewise, de Castilho Heiss et al. [5] noted that dietary inclusion of 250, 500, or 1000 g/ton polyphenol blend (polyphenols, flavonoids, and tannins) did not affect pH_24h_, color (a*, b*, and L* values), water retention capacity, drip loss, thawing loss, and cooking loss in pectoral muscle. Gao et al. [41] observed that the addition of epigallocatechin gallate (750 mg/kg) increased the L* value, decreased pH_45min_, pH_24h_, and drip loss, and did not influence the a* value, b* value, shear force, and cooking loss in the breast meat of broilers fed a high-fat diet. At the level of gene expression, dietary epigallocatechin gallate enhanced the expression of meat tenderness-related genes (*Co13a1*, *PGC-1α*, *FOXO1*, *GPAT*, *ADSL*, and *AIRC*) in pectoral muscle [41]. These results are in correspondence with Xiong et al. [42], who showed that dietary gallic acid (450 mg/kg diet) supplementation reduced pH_45min_ and drip loss, but had no significant impact on pH_24h_, color (a*, b*, and L* values), and shear force of breast muscle. They also documented that dietary gallic acid improved the gene expression of myosin heavy chain (*MyHC I* and *MyHC IIa*) in the breast muscle but reduced the gene expression of *MyHC IIx*, suggesting improved muscle fiber composition. It might be speculated that natural antioxidants (e.g., polyphenols, flavonoids, carotenoids) might be used in broiler diets to enhance breast and leg meat physical characteristics and might partially replace synthetic vitamin E. These bioactive compounds may influence gene expression of meat tenderness and myosin heavy chain, leading to better muscle development and overall stability.

**Figure 5:**
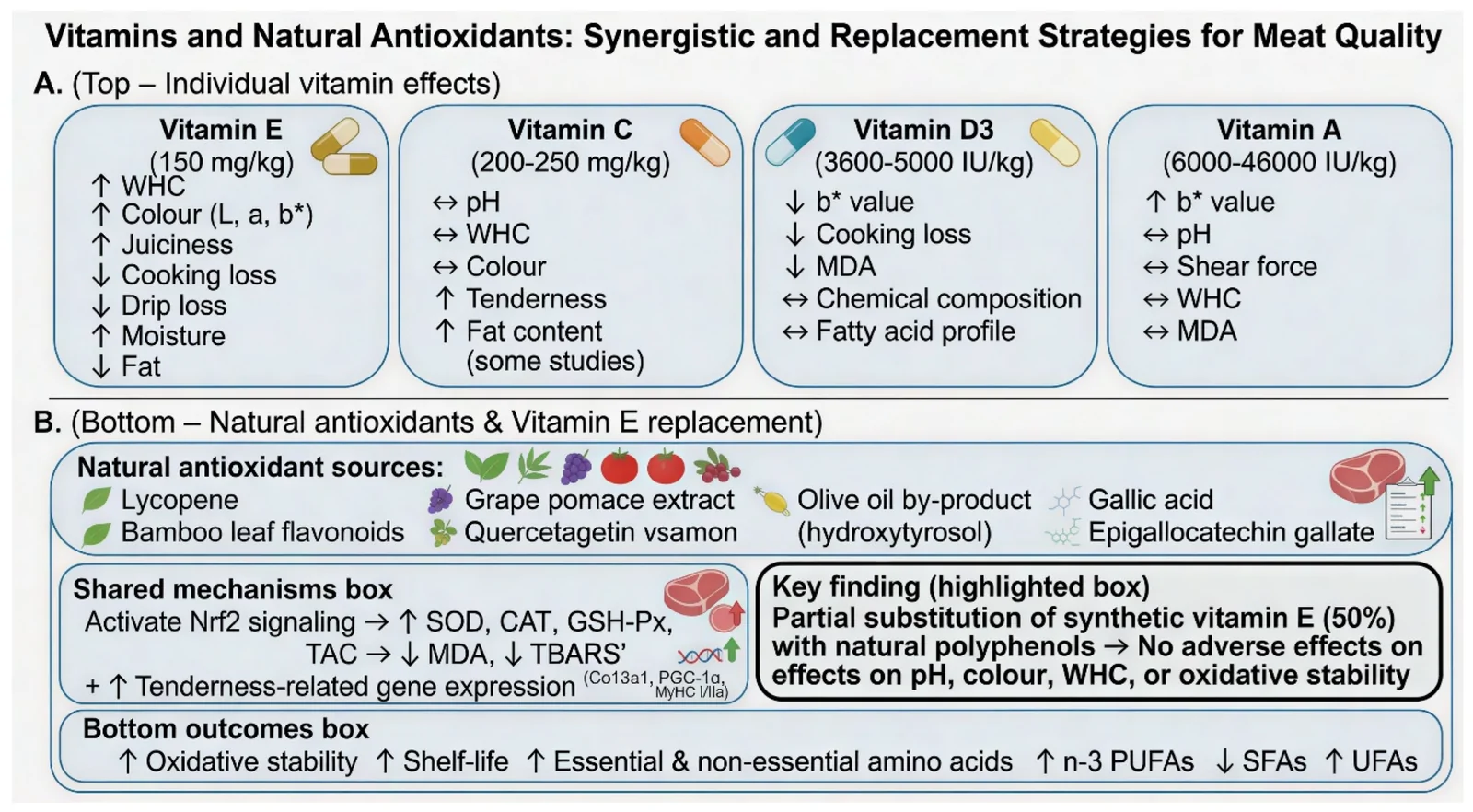
Dual-panel conceptual diagram showing the effects of vitamins and natural antioxidants with partial vitamin E replacement strategies on broiler meat quality (This figure was created by the authors using Microsoft PowerPoint and BioRender.com).

Regarding meat proximate composition, Andreaki et al. [89] indicated that partial substitution of synthetic vitamin E (50%) with 50 mg polyphenols as vinification by-products/kg feed from grape pomace extract decreased SFAs and the SFA/UFA ratio, increased MUFAs and UFAs, while having no significant effect on PUFAs and TAC in the breast muscle. Similarly, Corrales et al. [47] pointed out that substitution of 40 mg vitamin E/kg with 22.5 mg olive oil by-product extract rich in hydroxytyrosol/kg + 10 mg vitamin E/kg had no significant effect on MDA concentrations after 3 and 7 d of storage in the breast muscle. Ahsan et al. [90] postulated that dietary supplementation of a 60 mg/kg natural antioxidant blend (capsaicin, glucosinolates, saponins, terpenes, and curcumin) improved meat oxidative stability in the form of reducing TBARS and carbonyl groups in fresh and freeze–thawed breast meat after 30, 60, and 90 d of storage. Wu et al. [93] pointed out that the addition of 30 mg/kg dietary lycopene to the broilers’ feed had no significant effect on meat chemical composition (total moisture, CP, and EE content), increased concentrations of GSH-Px, SOD, and TAC; however, it reduced the concentration of MDA in leg muscle. de Castilho Heiss et al. [5] noted that dietary inclusion of 250, 500, or 1000 g/ton polyphenol blend (polyphenols, flavonoids, and tannins) decreased n−3PUFAs and had no significant effect on SFAs, UFAs, MUFAs, PUFAs, n−6PUFAs, UFA/SFA ratio, PUFA/SFA ratio, and n-3/n-6 ratio in the breast muscle. Likewise, Serra et al. [94] reported that dietary 2 g/kg of polyphenols (Oxilem^®^ or OxiGem^®^) increased CP and had no significant effect on moisture, ash, total lipids, and cholesterol contents in the breast muscle. Also, dietary treatments increased linoleic acid (C18:2 n-6) and γ-linolenic (C18:3 n-6) content in the breast muscle, while SFAs, UFAs, MUFAs, PUFAs, n−3PUFAs, n−6PUFAs, n-3/n-6 ratio, and TBARS were not affected. Gao et al. [41] observed that the addition of epigallocatechin gallate (750 mg/kg) improved the incidence of both essential amino acids (arginine, histidine, leucine, phenylalanine, threonine, lysine, and methionine) and non-essential amino acids (alanine, tyrosine, proline, and glutamic acid), as well as enhanced the activities of SOD and CAT, but the concentration of MDA was reduced in the breast meat of chickens fed a high-fat diet. Moreover, at the level of gene expression, dietary epigallocatechin gallate enhanced the expression of *SOD* mRNA in the breast meat [41]. Xiong et al. [42] showed that dietary gallic acid (450 mg/kg diet) supplementation increased docosahexaenoic acid (C22:6n-3) concentration and had no significant effect on SFAs, PUFAs, or the n-3/n-6 ratio in the breast muscle. They also postulated that dietary gallic acid increased activities of GSH-Px, SOD, and CAT and reduced MDA concentration in the breast meat. Cao et al. [91] elucidated that dietary bamboo leaf flavonoids (50 or 250 mg/kg diet) changed 14 metabolites, including flavonoids, amino acids, organic acids, phenolic compounds, and aromatic compounds in the breast muscle, which were involved in enhancing the activities of SOD and GSH-Px, while reducing MDA levels in the breast muscle. However, it should be noted that bamboo leaf flavonoids are derived from bamboo species (e.g., *Phyllostachys* spp.) that are cultivated mainly in East Asia, and standardized commercial extracts are not yet widely available on the global feed-additive market. Given this regional confinement, limited large-scale commercial production, and the relatively high extraction and processing costs compared with more established phytogenic antioxidants, the practical adoption of bamboo leaf flavonoids in broiler diets on a global scale may currently be constrained. Further cost–benefit and supply-chain feasibility studies are needed before this additive can be broadly recommended for the poultry industry. These results were confirmed by Hu et al. [92], who demonstrated that dietary natural flavonoid quercetagetin (10 mg/kg) had no significant effect on moisture, CP, EE, crude ash, umami amino acids, bitter amino acids, and sweet amino acids in the breast meat. They postulated that dietary quercetagetin enhanced meat oxidative stability (improved SOD, CAT, and TAC concentrations and minimized concentrations of MDA, 8-OHdG, and AOPPs) via modulating redox status and the Nrf2/ferroptosis signaling pathway. It might be mentioned that natural antioxidants might have a positive effect on meat oxidative stability via enhancing the protective antioxidative enzymes’ activities and minimizing lipid oxidation primarily in breast (*Pectoralis major*) muscle of broiler meat, biochemical outcomes consistent with, though not a direct measure of, extended shelf life, and nutritional quality (Figure 5).

## 11. Strategic and Synergistic Combinations of Functional Feed Additives

Although the preceding sections addressed each additive class separately for clarity, commercial broiler nutrition rarely relies on a single functional additive. Instead, feed formulators increasingly combine complementary additive classes to achieve synergistic, rather than merely additive, effects on gut health, antioxidant status, and meat quality, while also improving cost-effectiveness at industrial scale. Synbiotic formulations represent the most established example of intentional additive pairing, combining selected probiotic strains with a compatible prebiotic substrate to enhance probiotic viability, colonization, and metabolic activity beyond what either component achieves alone, which is introduced in detail in Section 5 of the present review.

Organic acid–phytogenic blends are similarly gaining commercial traction, pairing the gut-acidifying and antimicrobial action of organic acids with the antioxidant and flavor-enhancing properties of essential oils or plant bioactives. Tellini et al. [7] demonstrated that a microencapsulated blend of calcium formate, citric acid, and fumaric acid combined with orange essential oil, thymol, and carvacrol improved lipid oxidative stability (reduced MDA, enhanced DPPH/ABTS activity) without compromising physical meat traits, illustrating a synergistic interaction between acidifiers and phytogenic antioxidants in stabilizing muscle lipid peroxidation. Likewise, Akaichi et al. [63] reported that combining humic acid with organic acids improved pH_24h_, WHC, and sensory attributes beyond effects typically observed with either component alone. Galli et al. [66] showed that microencapsulation further protects organic acid–phytogenic actives through the gastrointestinal tract, improving delivery efficiency and fatty acid profile outcomes.

Vitamin–mineral matrices constitute a third practically important combination strategy, since vitamins E and C and organic/nano trace minerals (particularly Se and Zn) act as complementary components of the endogenous antioxidant network. Vitamins act as chain-breaking radical scavengers, and minerals act as essential cofactors of GSH-Px, SOD, and CAT. Pečjak et al. [46] combined vitamin E, vitamin C, and organic Se and observed elevated α-tocopherol and Se tissue concentrations with reduced MDA content, while De Grande et al. [86] reported that vitamin E supplementation, either alone or combined with organic Zn, reduced drip and thawing loss in the breast meat, consistent with the manuscript’s own earlier description of this study (Section 9); the published design did not statistically differentiate the magnitude of the effect between the single-nutrient and combined treatment arms, so a superior effect of the combination specifically cannot be concluded from this study. However, the original study did not report a significant interaction term or a direct pairwise comparison between the single-nutrient (vitamin E alone) and combined (vitamin E + organic Zn) treatment arms; consequently, whether the combined treatment conferred an additional benefit beyond vitamin E supplementation alone cannot be confirmed from the published data, and this finding should be regarded as evidence of compatibility between vitamin E and organic Zn rather than demonstrated synergism. Similarly, Alhasani [80] found that SeNPs, ZnNPs, and their combination (1.5 mg/kg SeNPs + 2.0 mg/kg ZnNPs) produced directionally consistent changes in meat physical and oxidative parameters (increased pH, L*, and b* values; reduced tenderness) regardless of whether the nanoparticles were administered singly or in combination. As no statistically significant interaction term or direct pairwise comparison between the single-nanoparticle and combined treatments was reported, these data do not support an interactive or synergistic relationship between SeNPs and ZnNPs, and the comparative language used in the original description of this study has been revised accordingly.

A related and commercially relevant strategy is the partial replacement of synthetic vitamin E with natural antioxidant sources (e.g., polyphenols, hydroxytyrosol-rich extracts), as shown by Andreaki et al. [89] and Corrales et al. [47], where 50% substitution maintained meat quality and oxidative stability while reducing reliance on synthetic vitamin inputs—an approach with direct implications for feed cost and “clean-label” positioning in commercial production. Moreover, Ahsan et al. [90] evaluated a natural antioxidant blend administered alone or in combination with the probiotic *Bacillus licheniformis* and reported sustained reductions in TBARS and protein carbonyls across extended (30–90 d) storage in the treatments assessed (a duration more representative of retail shelf-life conditions than most single-additive trials). However, as noted in the manuscript’s own earlier description of this study (Section 10), a statistically superior effect of the combined treatment over the antioxidant blend alone was not reported. So, this study is better regarded as an example of a cross-class additive combination with no demonstrated adverse interaction, rather than confirmed cross-class synergy. Moreover, as the original study was not confirmed to report a significant interaction term or a direct pairwise comparison between the natural-antioxidant-alone and combined treatment arms, this finding is presented here as evidence of compatibility between the natural antioxidant blend and B. licheniformis under extended storage, rather than as confirmed cross-class synergy; the term “synergy” has accordingly been removed from this description.

Collectively, these findings indicate that the strategic pairing of additive classes—synbiotics, organic acid–phytogenic complexes, and vitamin–mineral matrices—can produce more robust, consistent, and commercially translatable improvements in meat physicochemical quality, nutritional value, and oxidative stability than single additives evaluated in isolation. This is mechanistically plausible given that these combinations typically target complementary pathways (e.g., gut microbiota modulation plus Nrf2-mediated antioxidant gene expression, or radical scavenging plus enzymatic cofactor supply) rather than a single mode of action. However, dose ratios, encapsulation technology, and strain/extract compatibility require further systematic optimization, and head-to-head trials comparing combined versus single-additive strategies under identical commercial conditions remain limited and are recommended for future research.

## 12. Limitations

Several limitations should be considered when interpreting the findings of this review. First, substantial heterogeneity existed among the reviewed studies with respect to additive source and chemical form, strain or botanical origin, inclusion level, duration of supplementation, basal diet composition, bird genotype, age, sex, rearing conditions, muscle examined, storage conditions, and the analytical methods used to assess meat quality. These differences limit direct quantitative comparisons among studies and may partly explain the variable responses observed for pH, color, water-holding capacity, chemical composition, and oxidative stability. Second, although the review was focused on broilers, a limited number of studies involved other poultry production categories or genetically distinct chicken populations. For example, some mechanistic evidence was derived from laying hens, whereas other meat-quality findings were obtained from slow-growing or indigenous chickens. Such evidence may provide useful biological context but should not be assumed to be directly transferable to fast-growing commercial broilers without further validation. Therefore, conclusions regarding mechanisms and the practical efficacy of individual additives should be interpreted within the species, genotype, production system, and experimental conditions in which the original evidence was generated. Third, the present review primarily evaluated meat physicochemical characteristics, nutritional composition, fatty acid profile, and biochemical indicators of oxidative stability. Several studies reported reductions in TBARS or MDA and improvements in antioxidant capacity; however, these biochemical endpoints should not be interpreted as direct evidence of prolonged commercial shelf life unless supported by appropriate storage studies incorporating relevant microbiological, sensory, and/or spoilage endpoints. Similarly, food safety in the specific contexts of pathogen load, chemical residues, and toxicological risk was not comprehensively assessed across the reviewed studies. Therefore, improvements in meat quality or oxidative stability should not be equated with demonstrated improvements in overall food safety. Finally, the available evidence remains insufficient to establish universally applicable inclusion rates or definitive synergistic combinations for all functional feed additives. Differences in additive formulation, dose, matrix interactions, and production conditions make it difficult to extrapolate individual experimental results directly to commercial practice. Further standardized, adequately powered studies using comparable genotypes, dietary backgrounds, additive specifications, storage protocols, and food-safety endpoints are warranted to establish more robust dose–response relationships, validate proposed mechanisms, and determine the practical and safety implications of combined functional feed-additive strategies.

## 13. Conclusions

It might conclude that supplementation of functional feed additives into broiler diets represented a sophisticated nutritional strategy to meet consumer demands for high-quality and nutritionally favorable poultry meat. It should be clarified that food safety in the sense of pathogen load, chemical residues, or toxicological risk was not assessed in the studies reviewed here, and no claim regarding meat safety in this sense should be inferred from the physicochemical and oxidative stability findings summarized in this review; such claims would require dedicated microbiological, residue, and toxicological evaluation. Dietary supplementation of probiotics, prebiotics, phytobiotics, trace minerals, and natural antioxidants most consistently improved specific physical meat traits—namely WHC, cooking loss, and shear force/tenderness—although effects on pH and color were more variable across studies within these same additive classes. Synbiotics, organic acids, and vitamins generally showed no adverse effects on physical meat characteristics; however, this should not be read as evidence of uniformly positive or consistent benefit, since several individual traits within these categories, particularly pH, color indices, and shear force, showed mixed or directionally divergent responses across studies. Probiotics, prebiotics, synbiotics, and trace minerals improved the basic meat chemical composition, while organic acids and phytobiotics had no negative effects on meat proximate composition. It is noteworthy to indicate that dietary probiotics, prebiotics, synbiotics, phytobiotics, organic acids, trace minerals, vitamins, and natural antioxidants modified meat lipid profile toward healthy fats and enhanced oxidative stability, as evidenced by reduced TBARS/MDA and increased antioxidant enzyme activity across the reviewed studies; these biochemical findings are consistent with, though generally not accompanied by, directly measured shelf-life trials with defined storage endpoints. Additionally, natural antioxidants such as flavonoids and lycopene showed promise in partially replacing synthetic vitamins. Importantly, evidence reviewed here suggests that strategic, matrix-based combinations of these additive classes—synbiotics, organic acid–phytogenic blends, and vitamin–mineral matrices—more closely reflect commercial-scale poultry nutrition practice and may deliver more consistent and synergistic benefits than single additives, through complementary modulation of gut microbiota, antioxidant enzyme systems, and lipid metabolism. While the functional feed additives generally had a positive or neutral impact on meat quality in broilers, the variability in the results obtained across studies suggested that further studies are still required to determine the optimal dosages, chemical forms, and synergistic combinations to obtain benefits for the poultry industry. Future research should therefore prioritize dedicated dose-titration trials to establish practical inclusion recommendations, safety and residue assessments for novel or nanoparticle-based additives, and factorial or combinatorial trial designs capable of directly testing the synergy and substitution hypotheses raised in this review, before such strategies can be translated into firm commercial guidance for the poultry industry.

## Data Availability

The original contributions presented in this study are included in the article. Further inquiries can be directed to the corresponding author.
